# Genetically encoded calcium indicator with NTnC-like design and enhanced fluorescence contrast and kinetics

**DOI:** 10.1186/s12896-018-0417-2

**Published:** 2018-02-13

**Authors:** D. A. Doronin, N. V. Barykina, O. M. Subach, V. P. Sotskov, V. V. Plusnin, O. A. Ivleva, E. A. Isaakova, A. M. Varizhuk, G. E. Pozmogova, A. Y. Malyshev, I. V. Smirnov, K. D. Piatkevich, K. V. Anokhin, G. N. Enikolopov, F. V. Subach

**Affiliations:** 1 Moscow Institute of Physics and Technology, Moscow, 123182 Russia; 2 P.K. Anokhin Institute of Normal Physiology, Moscow, 125315 Russia; 3 National Research Center “Kurchatov Institute”, Moscow, 123182 Russia; 4 Lomonosov Moscow State University, Moscow, 119991 Russia; 5 Federal Research and Clinical Center of Physical-Chemical Medicine of Federal Medical Biological Agency, Moscow, 119435 Russia; 6 Engelhardt Institute of Molecular Biology RAS, Moscow, 119991 Russia; 7 Institute of Higher Nervous Activity and Neurophysiology of RAS, Moscow, 117485 Russia; 8 MIT Media Lab, Massachusetts Institute of Technology, Cambridge, MA USA; 9 Department of Anesthesiology, Stony Brook University Medical Center, Stony Brook, NY 11794 USA; 10 Center for Developmental Genetics, Stony Brook University, Stony Brook, NY 11794 USA

**Keywords:** Calcium imaging, Genetically encoded calcium indicator, Protein engineering

## Abstract

**Background:**

The recently developed genetically encoded calcium indicator (GECI), called NTnC, has a novel design with reduced size due to utilization of the troponin C (TnC) as a Ca^2+^-binding moiety inserted into the mNeonGreen fluorescent protein. NTnC binds two times less Ca^2+^ ions while maintaining a higher fluorescence brightness at the basal level of Ca^2+^ in neurons as compared with the calmodulin-based GECIs, such as GCaMPs. In spite of NTnC’s high brightness, pH-stability, and high sensitivity to single action potentials, it has a limited fluorescence contrast (F^-Ca2+^/F^+Ca2+^) and slow Ca^2+^ dissociation kinetics.

**Results:**

Herein, we developed a new NTnC-like GECI with enhanced fluorescence contrast and kinetics by replacing the mNeonGreen fluorescent subunit of the NTnC indicator with EYFP. Similar to NTnC, the developed indicator, named iYTnC2, has an inverted fluorescence response to Ca^2+^ (i.e. becoming dimmer with an increase of Ca^2+^ concentration). In the presence of Mg^2+^ ions, iYTnC2 demonstrated a 2.8-fold improved fluorescence contrast in vitro as compared with NTnC. The iYTnC2 indicator has lower brightness and pH-stability, but similar photostability as compared with NTnC in vitro. Stopped-flow fluorimetry studies revealed that iYTnC2 has 5-fold faster Ca^2+^ dissociation kinetics than NTnC. When compared with GCaMP6f GECI, iYTnC2 has up to 5.6-fold faster Ca^2+^ association kinetics and 1.7-fold slower dissociation kinetics. During calcium transients in cultured mammalian cells, iYTnC2 demonstrated a 2.7-fold higher fluorescence contrast as compared with that for the NTnC. iYTnC2 demonstrated a 4-fold larger response to Ca^2+^ transients in neuronal cultures than responses of NTnC. iYTnC2 response in neurons was additionally characterized using whole-cell patch clamp. Finally, we demonstrated that iYTnC2 can visualize neuronal activity in vivo in the hippocampus of freely moving mice using a nVista miniscope.

**Conclusions:**

We demonstrate that expanding the family of NTnC-like calcium indicators is a promising strategy for the development of the next generation of GECIs with smaller molecule size and lower Ca^2+^ ions buffering capacity as compared with commonly used GECIs.

**Electronic supplementary material:**

The online version of this article (10.1186/s12896-018-0417-2) contains supplementary material, which is available to authorized users.

## Background

Genetically encoded calcium indicators (GECIs) are broadly used for in vivo visualization of neuronal activity associated with calcium transients in the living cells. Fluorescent protein-based GECIs can be classified into three major types by their design (Additional file 1: Figure S1).

The first type of sensors is represented by FRET (fluorescence resonance energy transfer)-based indicators, which are composed of two fluorescent proteins with a Ca^2+^-binding domain located between them [1]. For example, the Twitch family of FRET indicators has a minimal Ca^2+^-binding motif from the C-terminal domain of troponin C (TnC) isolated from the toadfish, *Opsanus tau* [2]. The advantage of the Twitch FRET indicators over the majority of other GECIs is the minimal number of calcium ion-binding sites per molecule that ensures less influence on the cellular concentration of free calcium ions [3]. While FRET GECIs are sensitive and specific to Ca^2+^ ions, their application is limited, in particular, because of expensive equipment required for their imaging, low fluorescence contrast, and their large molecular size.

The second type is circularly permutated FP (cpFP)-based GECIs which are consisted of one cpFP with CaM/M13-like peptide pair attached close to its chromophore (Additional file 1: Figure S1a) [4, 5]. As compared with FRET-based indicators they have advantageously smaller molecular size but their CaM/M13 indicatory domain binds twice as many calcium ions as the Twitch family of FRET indicators (i.e., four vs two calcium ions per molecule). Due to their low fluorescence at resting Ca^2+^ levels in neurons, most of the cpFP-based indicators require co-transfection with bright, spectrally distinct markers to facilitate hunting for the positive cells in vivo, which is not the case for FRET-based indicators [6].

To overcome the limitations of the first two types of GECIs, NTnC-like type of GECIs was recently engineered [7]. It combines the advantages of the sensory domain from the Twitch FRET indicators with the fluorescent domain of the cpFP-based indicators, which simultaneously reduces the number of Ca^2+^-binding sites as well as the molecular size of the indicator. For example, NTnC GECI has a size of 311 a.a., which is less than that for cpFP- and FRET-based indicators by 105 and 245 a.a., respectively. In addition, NTnC has a high brightness intensity and pH stability in comparison to cpFP GECIs. NTnC demonstrates an inverted phenotype; its fluorescence is quenched by the Ca^2+^ ions. Due to inverted fluorescent Ca^2+^ dependence, NTnC provides non-active neurons or cells with low resting calcium concentrations to be fluorescently visible; thus, eliminating the need for co-expression of bright fluorescent cell marker. In addition, NTnC has been previously applied for in vivo imaging of neuronal activity in awake mice [7]. Despite the mentioned advantages, NTnC has one main limitation, which is a 2-fold decrease of fluorescence contrast (F^-Ca2+^/F^+Ca2+^) and slower Ca^2+^ dissociation kinetics in comparison to GCaMP6s.

Based upon another green fluorescent GECI, such as Camgaroo1 [8], with an 8-fold higher fluorescence contrast, we presumed that the replacement of fluorescent subunit of NTnC with another fluorescent protein may result in an indicator with enhanced contrast and improved kinetics. Through this reasoning, we have developed a new single-fluorophore-based GECI with two Ca^2+^-binding sites utilizing the NTnC-like design, but with enhanced fluorescence contrast and kinetics. This indicator, named iYTnC2, uses tsTnC as the Ca^2+^-binding moiety, inserted within the EYFP fluorescent protein. iYTnC2 shows an inverted response to Ca^2+^ ions, a 4-fold higher fluorescence contrast, a 6-fold lower brightness, and a similar photostability when compared to NTnC in vitro. According to stopped-flow fluorimetry studies, iYTnC2 dissociates from Ca^2+^ ions 5-fold faster than NTnC or 1.7-fold slower than GCaMP6f. iYTnC2 has Ca^2+^ association kinetics of up to 5.6-fold faster than GCaMP6f. During Ca^2+^ transients in mammalian cells, iYTnC2 demonstrated fluorescence contrast 2.7-fold higher than the NTnC indicator. In neuronal cultures, iYTnC2 demonstrated a 4-fold higher fluorescence contrast than the NTnC indicator. We additionally characterized iYTnC2 behavior in neurons using patch clamp. In spite of its 6-fold lower brightness when compared to NTnC in vitro, iYTnC2 could be used to visualize neuronal activity in vivo in the mouse brain using miniaturized nVista microscope.

## Methods

### Mutagenesis and library screening

Primary construction of indicators and directed saturated mutagenesis of linkers between fluorescent and indicatory parts were accomplished using polymerase chain reaction (PCR) with overlapping fragments [9]. For PCR amplification, we used a С1000 Touch Thermal Cycler (Bio-Rad, USA). Random mutations were introduced over the whole length of the indicator gene using PCR in the presence of manganese ions with conditions to achieve 2–3 random mutations per 1000 bp (according to the Diversify PCR Random Mutagenesis Kit User Manual, Clontech, USA).

The genes of the indicators were cloned using the BglII/EcoRI restriction sites of the pBAD/HisB-TorA plasmid (Invitrogen, USA) encoding the TorA signal sequence, which is necessary for the transport of indicators into the periplasmic space of bacteria, and transformed these plasmids into electrocompetent BW25113 bacteria as described in reference [7].

Screening of bacterial libraries was performed sequentially on Petri dishes, bacterial suspensions in 96-well plate format, and purified proteins as described in reference [7].

### Protein purification and characterization

The bacterial cultures carrying pBAD/HisB-TorA-indicator plasmid were grown in LB medium supplemented with 0.002% arabinose and 100 μg/ml ampicillin overnight at 37 °C and 220 rpm. The cultures were then centrifuged at 4648 g for 10 min, and the cell pellets were resuspended in PBS at pH 7.4 with 300 mM NaCl and lysed by sonication on ice. The recombinant proteins were purified using Ni-NTA resin (Qiagen, USA), followed by dialysis for 12–16 h against buffer solutions (30 mM HEPES or 10 mM Tris-HCl, 100 mM KCl, pH 7.2, supplemented with either 10 mM EDTA or 10 mM CaCl_2_ or without EDTA and CaCl_2_). The absorbance values and excitation and emission spectra were measured with a CM2203 spectrofluorometer (Solar, Belarus).

Chromophore extinction coefficients for purified iYTnC and iYTnC2 in the Ca^2+^-saturated states were measured in buffer 10 mM Tris-HCl, 100 mM KCl, 10 mM CaCl_2_, pH 7.2, by alkaline denaturation with 1 M NaOH and using extinction coefficients for GFP-like chromophores equal to 44,000 M^− 1^ cm^− 1^ in 1 M NaOH [10]. Extinction coefficients for purified iYTnC and iYTnC2 in the Ca^2+^-free states were calculated in buffer 10 mM Tris-HCl, 100 mM KCl, 10 mM EDTA, pH 7.2 by comparing their absorption spectra with absorption spectra for iYTnC_sat_ and iYTnC2_sat_ with the same absorbance at 280 nm.

For quantum yield determination, the integrated fluorescence values of purified iYTnC in the Ca^2+^-free and Ca^2+^ saturated states were measured in buffer (10 mM Tris-HCl, 100 mM KCl, pH 7.2) supplemented with either 10 mM EDTA or 10 mM CaCl_2_, respectively as previously reported [11].

For equilibrium K_d_ determination, the two stock buffers 30 mM HEPES, 100 mM KCl, pH 7.2 containing 10 mM EGTA (zero free Ca^2+^) or 10 mM Ca-EGTA (39 μM free Ca^2+^) were mixed in various ratios to give 15 solutions with different free Ca^2+^ concentrations, as described previously [12]. The purified iYTnC protein in buffer 30 mM HEPES, 100 mM KCl, pH 7.2 was added (1:100, till final concentration of 2 μg/ml) to prepared solutions with different free Ca^2+^ concentrations and after 20 min of equilibration at r.t. its green fluorescence was measured on Modulus™ II Microplate Reader (TurnerBiosystems, USA). Three replicates were averaged for analysis. The calculated K_d_ represents the concentration of Ca^2+^ when the fluorescence change of the indicator is half of its maximum value. Titration of iYTnC to Ca^2+^ ions in the presence of Mg^2+^ was performed in the same manner except for two stock buffers 30 mM HEPES, 100 mM KCl, 1 mM MgCl_2_, pH 7.2 containing 10 mM EGTA (zero free Ca^2+^) or 10 mM Ca-EGTA (39 μM free Ca^2+^) were mixed.

Size-exclusion chromatography was performed with a SuperdexTM 75 10/300 GL column using GE AKTA Explorer (Amersham Pharmacia, UK) FPLC System.

In addition, pH titration, photobleaching experiments, and protein maturation were performed as described in [7].

### Stopped-flow fluorimetry

Ca^2+^-binding kinetics experiments were performed on a Chirascan Spectrofluorimeter (Applied Photophysics, UK) equipped with a stopped-flow module at 20 °C. Fluorescence excitation was set at 493 nm, and fluorescence emission was collected using a 515 nm cut-off filter. Three replicates were averaged for analysis. Kinetic records were fitted to either a single or a double exponential using DataFit9 (Oakdale Engineering, USA).

To measure association kinetics, iYTnC or control GCaMP6f (20 μg/ml) in 30 mM HEPES buffer (pH 7.2) containing 100 mM KCl and 1 mM EGTA was rapidly mixed (1:1) with 30 mM HEPES buffer (pH 7.2) containing 100 mM KCl, 10 mM EGTA and increasing Ca^2+^ concentrations. Exponential fitting of the fluorescence signal changes over time provided the observed association rate constants (k_obs_). Fitting the observed data to the equation k_obs_ = k_on_ × [Ca^2+^]^n^ + k_off_ provided the association rate constant (k_on_) and Hill coefficient (n). K_d kinetic_ = (k_off_/k_on_)^1/n^.

To measure dissociation kinetics, protein solution (20 μg/ml) in 30 mM HEPES (pH 7.2), 100 mM KCl and 1 μM CaCl_2_ was rapidly mixed (1:1) with 30 mM HEPES (pH 7.2), 100 mM KCl, and 10 mM EGTA. Exponential fitting of the fluorescence signal changes over time provided dissociation rate constants (k_off_).

### Cell culture and transfection

HeLa Kyoto or HEK293T cell lines were maintained in Dulbecco’s Modified Eagle Medium (DMEM) (GIBCO) supplemented with 10% fetal bovine serum (FBS) (Sigma), 50 U/ml penicillin, and 50 μg/ml streptomycin (GIBCO). Plasmids for transfection were prepared using a Plasmid Miniprep purification kit (Evrogen, Russia). Transfection was performed using TurboFectTM (Thermo Fisher Scientific, USA) according to the manufacturer’s protocol.

### Mammalian plasmid construction

In order to construct the pAAV-*CAG*-NES-mCherry plasmid, NES-mCherry gene was PCR amplified as the BamHI-BsrGI fragment and swapped with the iRFP-P2A-EGFP gene in the pAAV-*CAG*-iRFP-P2A-EGFP vector. In order to construct pAAV-*CAG*-NES-iYTnC and pAAV-*CAG*-NES-R-GECO1 plasmids, iYTnC and R-GECO1 were PCR amplified as the BglII-EcoRI fragments and swapped with the mCherry gene in the pAAV-*CAG*-NES-mCherry vector.

### rAAV particle production and isolation

The rAAV particles were purified as described previously [7].

### Mammalian live-cell imaging

HeLa Kyoto cell culture was imaged 24–48 h after transfection using a laser spinning-disk Andor XDi Technology Revolution multi-point confocal system (Andor, UK) equipped with an inverted Nikon Eclipse Ti microscope, a 75 W mercury-xenon lamp (Hamamatsu, Japan), a 60× oil immersion objective NA 1.4 (Nikon, Japan), a 16-bit QuantEM 512SC electron-multiplying CCD (Photometrics, USA), and a cage incubator (Okolab, Italy). Before imaging, the culture medium was changed to Dulbecco’s Phosphate Buffered Saline (DPBS) buffered with 20 mM HEPES, pH 7.4.

For time-lapse imaging experiments with varying Ca^2+^ concentration, 1 mM EDTA and 2.5 μM ionomycin were added to cells for imaging calcium indicators in the Ca^2+^-free state. After imaging calcium indicators in the apo-state, cells were washed with DPBS buffered with 20 mM HEPES, pH 7.4. Next, 2 mM CaCl_2_ and 2.5 μM ionomycin were added to induce fluorescence signal for Ca^2+^-saturated calcium indicators.

### Isolation, transduction, and imaging of neuronal cultures

Dissociated neuronal cultures were isolated from four C57BL/6 mice at postnatal days 0–3. For euthanasia of P0–3 neonates, we used decapitation by sharp scissors. The neuronal cultures were grown on 35-mm MatTek glass-bottom dishes in Neurobasal Medium A (GIBCO, UK) supplemented with 2% B27 Supplement (GIBCO, UK), 0.5 mM glutamine (GIBCO, UK), 50 U/ml penicillin, and 50 μg/ml streptomycin (GIBCO, UK). On the 4th day in vitro, neuronal cells were transduced with 1–2 μl rAAV viral particles carrying AAV-*CAG*-NES-iYTnC, AAV-*CAG*-NES-R-GECO1, or AAV-*CAG*-NTnC. Cells were imaged using an Andor XDi Technology Revolution multi-point confocal system.

### Whole-cell electrophysiology and calcium imaging

Whole-cell recordings with patch electrodes were made from cultured neurons expressing GECIs. Cells were selected under visual control using standard filter sets for green and red fluorescence and DIC infrared video microscopy. The patch electrodes were filled with a potassium gluconate-based solution (130 mM potassium gluconate, 20 mM KCl, 4 mM Mg-ATP, 0.3 mM Na_2_-GTP, 10 mM sodium phosphocreatine, 10 mM HEPES at pH 7.3) and had a resistance of 6–8 MΩ. During recording, cells were bathed in modified Hank’s solution containing: 138 mM NaCl, 1.26 mM CaCl_2_, 0.5 mM MgCl_2_, 0.4 mM MgSO_4_, 5.3 mM KCl, 0.44 mM KH_2_PO_4_, 4.16 mM NaHCO_3_, 0.34 mM Na_2_HPO_4_, 10 mM Glucose, 10 mM HEPES at pH 7.4 and r. t. Recordings were made with a MultiClamp 700B (Molecular Devices, USA) amplifier in bridge mode. After amplification and low-pass filtering at 10 kHz, data was digitized at 20 kHz and fed into a computer using the Digidata 1500 interface and pCLAMP software (Molecular Devices, USA). Cells were stimulated with 50 Hz trains of short (5 ms) intracellularly applied current pulses; the intensity of the pulses was adjusted to reliably induce action potentials for each cell.

Optical imaging was performed on an Olympus BX51WI microscope equipped with 40× water immersion objective, two camera ports, and collimated light emitting diodes (LED) with the peak emission wavelength of 470 nm (for iYTnC2 and GCaMP6s) and 530 nm (for R-GECO1) (Thorlabs, USA) for epi-illumination. Imaging was performed with a NeuroCCD camera (80 × 80 pixels, RedShirtImaging, USA) using a frame rate of 40 Hz. Fluorescence changes were measured with single wavelength excitation and emission > 510 nm for green and > 610 nm for red fluorescence. Analysis of optical data, including spatial averaging, high-pass and low-pass filtering, was conducted with the Neuroplex 7 software (RedShirtImaging, USA). The time-courses of the responses were corrected for bleaching using a linear regression computed through the mean values 2 s before the stimulation and by subtracting the extrapolated values.

### Animals and surgery for imaging with an nVista HD miniature microscope

Five adult male C57BL/6 mice, aged 20 weeks at the start of the experiments, were used for this study. Mice underwent two surgical procedures under zoletil-xylazine anesthesia (40 and 5 mg/kg, respectively). First, a circular 2-mm-diameter craniotomy was made, and 1 μl of rAAV viral particles (carrying AAV-*CAG*-NES-iYTnC2) was injected through a glass micropipette into the hippocampus (left hemisphere; stereotaxic coordinates: − 1.9 mm AP, − 1.4 mm ML to Bregma). All exposed surfaces of the brain tissue were sealed with KWIK-SIL silicone adhesive (WPI Inc., USA). Ten days later, the silicone was removed, and the dura mater was extracted from the craniotomy site. Then, a GLP 1040 lens probe (Inscopix Inc., USA) was lowered slowly to a depth of 1.1 mm while constantly washing the craniotomy site with sterile cortex buffer. Next, all the exposed tissue was sealed with KWIK-SIL and pink dental cement (Stoelting, USA). Afterwards animals were euthanized by lethal dose (0.1 ml/10 g) of 15% saline solution of chloral hydrate (Panreac, Spain).

### Ca^2+^ in vivo imaging with the nVista HD miniature microscope

After 1 week recovery period, mice were anesthetized, and baseplates for attaching the portable nVista HD miniature microscope (Inscopix Inc., USA) were mounted onto the dental acrylic caps. A few days after baseplate mounting, we sequentially attached the nVista HD microscope to awake mice that were then placed in open field arena. Five min-long calcium activity movies were captured at a frame rate of 20 Hz. Image analysis of the acquired data was performed using Mosaic software (Inscopix Inc., USA) and custom MATLAB scripts (Supplementary Methods in Additional file 2).

## Results

### Development of a novel green fluorescent calcium indicator with the NTnC-like design

To enhance fluorescence contrast of the NTnC GECI, we swapped its mNeonGreen fluorescent subunit with EYFP and performed several rounds of optimization using directed molecular evolution in a bacterial system. As a fluorescent component, we chose the EYFP protein because it has been previously used in GECI with a high fluorescence contrast, such as Camgaroo1 [8]. For the Ca^2+^-binding sensor subunit, we used a derivative of TnC from the toadfish, *Opsanus tau*, similar to the GECI, NTnC. First, we constructed three libraries with the insertion of TnC between residues 144 and 146 (L1), 144 and 145 (L2), or 147 and 149 (L3) of EYFP (according to EGFP enumeration) with the incorporation of a 3- or 2-amino-acid-long linker between the fluorescent and sensory components (Additional file 1: Figure S1b and Additional file 3: Figure S2).

The generated libraries were analyzed using two-step screening strategy. First, we performed imaging of a library of indicators targeted to the *E. coli* periplasm on Petri dishes before and after treatment with a buffer containing EDTA to select clones with the highest fluorescence ratio. Second, the selected clones were analyzed in bacterial suspension in a 96-well plate format. After the second step of screening, we found that clones from the L1 library exhibited both direct and inverted fluorescence responses with similar fluorescence contrasts of 2.8- and 2.5-fold which corresponded to 1.8- and 1.5-fold ΔF/F increase to NTnC, respectively.

The best clones having inverted phenotype from the L1 library were further subjected to the several rounds of random mutagenesis followed by the same two-step screening. During each round, we screened approximately 20,000–40,000 colonies to identify variants with the largest Ca^2+^-dependent changes in green fluorescence similar to that described above. Finally, a variant with the best performance selected after 4 rounds of the directed evolution, named iYTnC (inverse EYFP derived TnC-based calcium indicator), was characterized in vitro on purified protein (Additional file 4: Table S1, Additional file 5: Figure S3 and Additional file 6: Figure S4), in HeLa cells and on neuronal cultures (Supplementary Results in Additional file 7). In the presence of Mg^2+^ ions, iYTnC demonstrated 2-fold improved fluorescence contrast in vitro as compared with NTnC. During calcium transients in the cultured mammalian cells, iYTnC demonstrated 2.2-fold higher fluorescence contrast as compared with that for the NTnC. However, in cultured neurons during spontaneous activity, we observed no improvement in the fluorescent contrast of iYTnC over NTnC (Fig. 3a-c and Fig. 4a-c).

To address the low contrast of the iYTnC indicator in neurons, we attempted to develop its enhanced version via rational design strategy. Based upon a reduction of fluorescent contract and affinity to Ca^2+^ in the presence of Mg^2+^ ions (Additional file 4: Table S1), we decided to revert two mutations in the Ca^2+-^-binding domain, namely C155Y and D194G, that were randomly introduced into iYTnC during directed molecular evolution. Based on the location of these mutations in Ca^2+^-binding domain and the fact that the fluorescent contrast of NTnC was previously not affected by the Mg^2+^ ions (Additional file 3: Figure S2), we expected that reverting these mutations would eliminate the effect of Mg^2+^ on the fluorescent contrast of iYTnC in neurons. To avoid potential problems with the Cys155 residue oxidation and its post-translational modifications in mammalian cells, we replaced the Cys residue present in NTnC indicator with Ser in iYTnC, an amino acid with similar properties. We called this version as iYTnC2 and used it further for detailed characterization in vitro and in cultured cells. The iYTnC2 indicator had 21 mutations relative to the original template (Additional file 3: Figure S2).

### In vitro characterization of purified iYTnC2

**Fig. 1:**
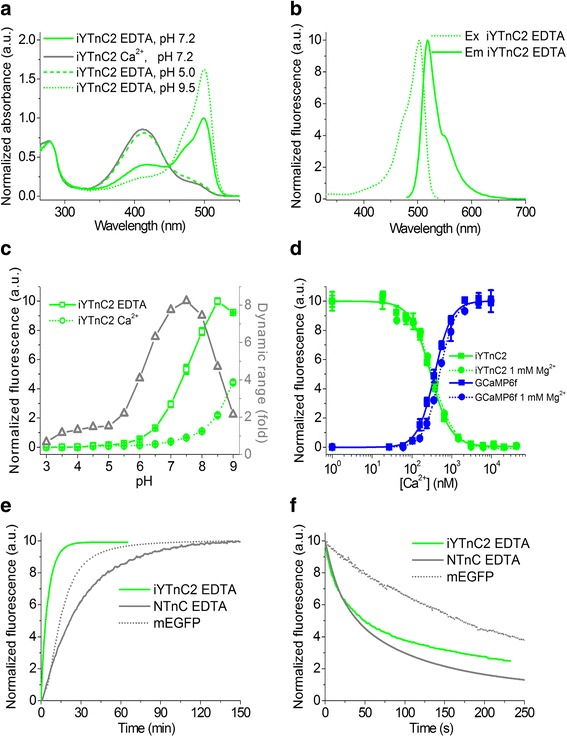
In vitro properties of the purified iYTnC2 indicator. **a** Absorbance spectra for iYTnC2 in Ca^2+^-bound or Ca^2+^-free states at indicated pH values. **b** Excitation and emission spectra for iYTnC2 in Ca^2+^-free state at pH 7.2. **c** Fluorescence intensity for iYTnC2 in Ca^2+^-free and Ca^2+^-bound states and their dynamic range as a function of pH. Error represents the standard deviation for the average of three records. **d** Ca^2+^ titration curves for iYTnC2 and GCaMP6f in the absence and in the presence of 1 mM MgCl_2_. **e** Maturation curves for iYTnC2, NTnC in Ca^2+^-free state, and mEGFP. **f** Photobleaching curves for iYTnC2, NTnC in Ca^2+^-free state, and mEGFP. The power of light before objective lens was 7.3 mW/cm^2^

The in vitro characteristics of the purified iYTnC2 calcium indicator are summarized in Table 1. At pH 7.2 in the Ca^2+^-free state iYTnC2_apo_ exhibited green fluorescence with emission peaks at 516 and 518 nm when excited at absorbance peaks at 413 and 499 nm, correspondingly. At pH 9.5, when iYTnC2_apo_ is in the deprotonated anionic fluorescent state, only one emission and one absorbance peaks were observed at 499 and 518 nm, respectively. At pH 5, when the chromophore of iYTnC2_apo_ protein is protonated, one emission and one absorbance peak were registered at 413 and 516 nm, respectively. In the Ca^2+^-saturated state iYTnC2_sat_ maximally absorbed at 410 nm and had dim fluorescence peaked at 520 nm (Fig. 1a, b). In both the Ca^2+^-free and Ca^2+^-saturated states, the brightness of the 410–413-nm absorbing forms of iYTnC2 was 11–20-fold lower than that of the main fluorescent 499-nm absorbing form. The brightness of the fluorescent 499-nm absorbing form of the iYTnC2_apo_ indicator in the Ca^2+^-free state was 6-fold lower than that for the control NTnC GECI (Table 1). The maximal fluorescence contrast of 8.1 ± 0.4-fold (through the text the reported values are mean ± standard deviation) for the iYTnC2 indicator between Ca^2+^-free and Ca^2+^-saturated states was in 4-fold larger than the contrast for the NTnC GECI.

**Table 1:** In vitro properties of purified iYTnC2 compared to NTnC

Properties		Proteins			
		iYTnC2		NTnC	
		apo	sat	apo	sat
Absorbance maximum (nm)		499 (413)	410	505	
Emission maximum (nm)		518 (516)	520	518	
Quantum yield^a^		0.33 ± 0.03 (0.041 ± 0.005)	0.033 ± 0.004	0.71 ± 0.05	0.65 ± 0.04
ε (mM^−1^ cm^− 1^)		37 ± 1 (14.8 ± 0.4)^c^	31.6 ± 0.7^b^	108 ± 6^b^	52 ± 1^c^
Brightness (%)		16 (0.8)	1.4	100	44
Fluorescence contrast (fold)	0 mM Mg^2+^	8.1 ± 0.4		2.0 ± 0.3	
	1 mM Mg^2+^	5.5 ± 0.2		2.0 ± 0.7	
p*K*a		7.4 ± 0.1	8.5 ± 0.1	6.09 ± 0.07	6.08 ± 0.02
K_d_ (nM)^d^	0 mM Mg^2+^	295 ± 9 [n = 1.7 ± 0.1]		84 ± 6 [n = 1.9 ± 0.1]	
	1 mM Mg^2+^	331 ± 22 [n = 1.6 ± 0.2]		192 ± 40 [n = 2.0 ± 0.4]	
K_d_^kin^ (nM)^e^		200 ± 150 [n = 1.9 ± 0.1]		94 ± 9 [n = 2.3 ± 0.1]	
k_on_ (s^−1^× M^-n^)^f^		6.2 ± 0.5 × 10^12^		6 × 10^15^	
k_off_ (s^−1^)^g^		1.12 ± 0.01		0.8 ± 0.1; 0.05 ± 0.01^h^	
t_1/2_^off^ (s)^g^		0.58 ± 0.01		3.00 ± 0.05	
Maturation half-time (min)^i^		3	ND	23	28
Photobleaching half-time (s)^j^		49 ± 19	ND	40 ± 8	70 ± 5

^a^QYs were determined at pH 7.20. GCaMP6s in the saturated state (QY = 0.61 [18]) and mTagBFP2 (QY = 0.64 [19]) were used as reference standards for 499- to 505- and dim fluorescent 410- to 416- nm absorbing states, respectively

^b^Extinction coefficients were determined by alkaline denaturation

^c^Extinction coefficients were estimated relative to iYTnC_sat_ or NTnC_apo_ with the same concentration determined according to the absorbance at 280 nm

^d^Hill coefficients are shown in square brackets. In the absence and the presence of 1 mM Mg^2+^ ions, GCaMP6f control GECI had K_d_ value of 370 ± 8 [n = 2.03 ± 0.08] and 492 ± 10 [n = 2.23 ± 0.09] nM, respectively

^e^K_d_^kin^, Hill coefficients and k_on_ values were obtained via fitting the observed association rates to the equation k_obs_ = k_on_ × [Ca^2+^]^n^ + k_off_ (Fig. 2a). K_d_^kinetic^ = (k_off_/k_on_)^1/n^. Hill coefficients are shown in square brackets. GCaMP6f has K_d_^kin^ value of 450 ± 300 nM [n = 2.4 ± 0.1]

^f^GCaMP6f has k_on_ value of (3.5 ± 2) × 10^15^ s^− 1^× M^-n^

^g^Refined k_off_ and t_1/2_^off^ values were determined from the dissociation kinetics records (Fig. 2d). GCaMP6f has t_1/2_^off^ and k_off_ values of 0.35 ± 0.02 s and 2.109 ± 0.002 s^− 1^, respectively

^h^Unlike iYTnC kinetics, NTnC kinetics do not agree with the two-state model. NTnC kinetic curves were fitted to double exponentials. k_off_ values were estimated from double exponential decay with individual exponent contributions of 0.48:0.52

^i^EGFP had a maturation half-time of 14 min

^j^mEGFP had a photobleaching half-time of 170 ± 20 s

First, we attempted to understand the factors that may contribute to the contrast of the iYTnC2 indicator. The iYTnC2 binding to calcium ions was accompanied by a 8.6-fold reduction in absorbance at 499 nm and increase in absorbance for the sensor at 410 nm. Absorbance contrast was practically the same as compared with the 8.1-fold fluorescence response (Table 1). Hence, binding of iYTnC2 to Ca^2+^ ions is accompanied by a transition from one form of the chromophore (with absorbance at 499 nm) into another form (with absorbance at 410 nm). Consequently, the high fluorescence contrast of the iYTnC2 indicator is related to the effective transition of 410-nm absorbing form into 499-nm one, the opposite to the NTnC indicator for which similar transition was incomplete [7].

Next, we characterized other important properties of the iYTnC2 GECI, dependences of its fluorescence and contrast from pH. The iYTnC2 exhibited shift in p*K*a values from 7.4 in the Ca^2+^-free state to 8.5 in the Ca^2+^-bound state (Fig. 1c, Table 1). As a result, the fluorescence of both states, as well as fluorescence contrast of iYTnC2, showed dependence on pH within the physiological range of pH 7–8. Similarly to iYTnC2, the commonly used GCaMP6s indicator based on pH-sensitive EGFP protein had also a pronounced pH-dependence of its ΔF/F response within the pH range of 5–8 [7]. In contrast, NTnC indicator based on pH-stable mNeonGreen FP had lower sensitivity to pH changes [7, 13]. Hence, sensitivity to pH was probably inherited by iYTnC2 and NTnC GECIs from their fluorescent progenitors.

We further assessed the affinity of the iYTnC2 indicator to Ca^2+^ ions to estimate ability of iYTnC2 to sense variations of calcium transients in neurons which typically vary between 50 and 10,000 nM [14, 15]. According to equilibrium binding titrations experiments, iYTnC2 demonstrated an equilibrium K_d_ value of 295 ± 9 nM which was 3.5-fold larger and practically similar to those values for NTnC and GCaMP6f, respectively (Fig. 1d and Table 1). Its affinity to Ca^2+^ ions was 1.2–2.0-fold weaker than those for the FRET indicators Twitch-1/2/3, which were based on the same TnC domain [2]. Probably mutations in the linkers and sensory subunits decreased the affinity of the iYTnC2 sensor to Ca^2+^ ions. Equilibrium Hill coefficient for the iYTnC2 indicator was lower than those for the NTnC and GCaMP6f GECIs evidencing decreased cooperativity of Ca^2+^ binding by iYTnC2.

We next characterized the response of iYTnC2 to Ca^2+^ ions under the conditions similar to the cytoplasm of neurons. In the presence of 1 mM Mg^2+^, purified iYTnC2 and NTnC demonstrated K_d_ values of 331 ± 22 and 192 ± 40 nM, respectively, which were in 1.1- and 2.3-fold larger than K_d_ values in the absence of Mg^2+^ ions (Fig. 1d and Table 1). At 1 mM Mg^2+^, a concentration that resembles that in neuronal cytoplasm, fluorescence contrast of iYTnC2 dropped to the value of 5.5 ± 0.2 which was 1.5-fold lower than in the absence of Mg^2+^ ions, but it was 2.8-fold higher than the contrast of the NTnC indicator in the same conditions.

We also characterized the oligomerization state of iYTnC2 and its maturation rate and photostability using NTnC and EGFP as the controls. In the presence of 5 mM Ca^2+^, purified iYTnC2 sensor at a concentration of 26 mg/ml eluted on size-exclusion chromatography as monomer (Additional file 8: Figure S5). At 37 °C, the iYTnC2 indicator in the Ca^2+^-free state matured till 50% for the 3 min and this process was 7.7- and 4.7-fold faster than for the NTnC and EGFP, respectively (Fig. 1e). Under a wide-field microscope equipped with a metal halide lamp, the iYTnC2 and NTnC indicators in the Ca^2+^-free state photobleached 3.5- and 4.2-fold faster than EGFP, respectively (Fig. 1f).

Overall, iYTnC2 demonstrated up to 4-fold higher fluorescence contrast, 6-fold dimmer brightness, lower pH stability, 3.5-fold decreased affinity to Ca^2+^ ions, monomeric state, faster maturation rate and similar photostability as compared with NTnC in vitro.

### Characterization of iYTnC2 calcium indicator kinetics using stopped-flow fluorimetry

**Fig. 2:**
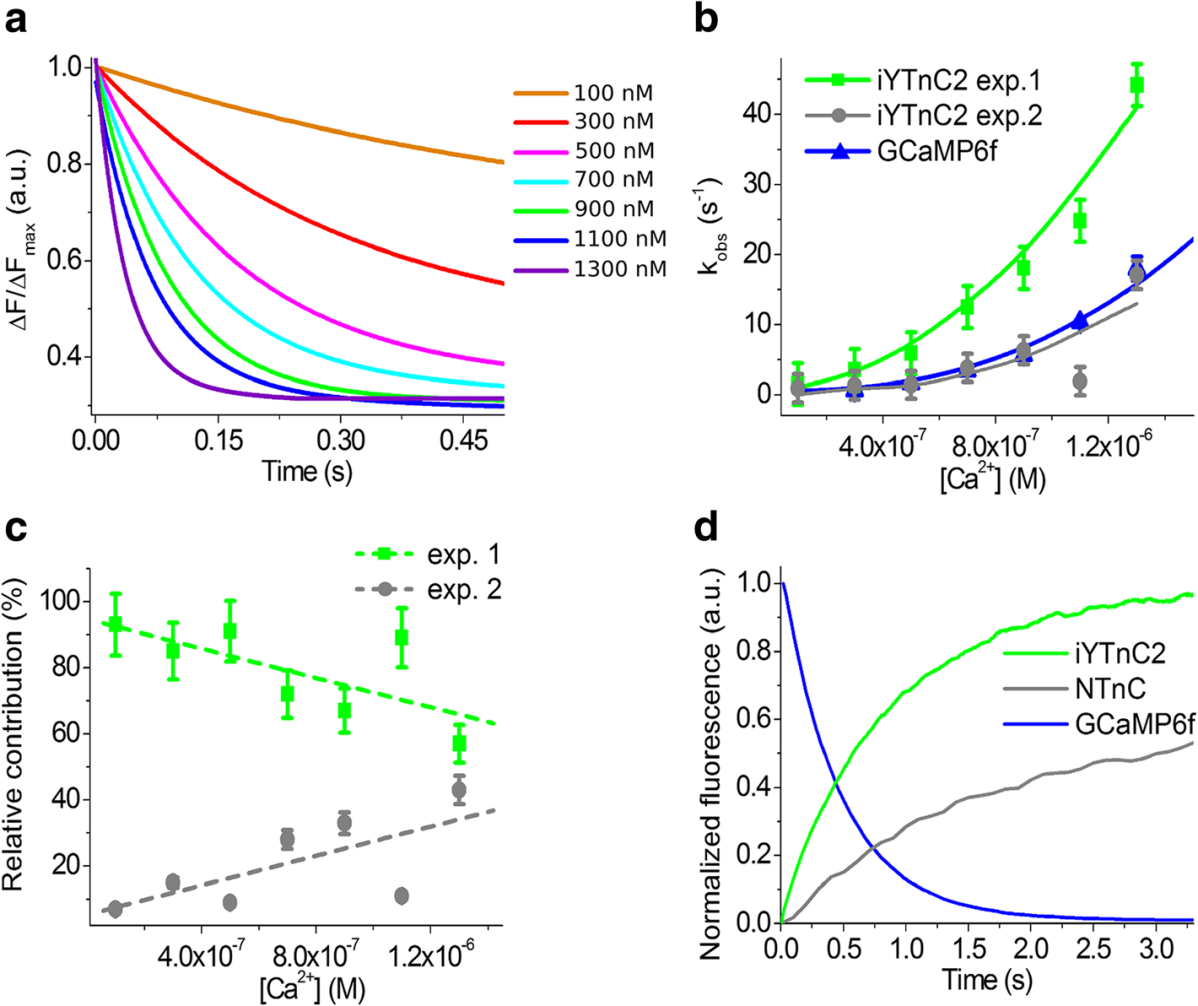
Calcium association and dissociation kinetics for the iYTnC2 and GCaMP6f indicators studied using stopped-flow fluorimetry. **a** Calcium association kinetics curves for iYTnC2. **b** Observed Ca^2+^ association rate constants determined from association curves for iYTnC2 and control GCaMP6f GECIs. For the iYTnC2 indicator, fast (green) and slow (grey) exponents are shown. **c** Relative contribution of monoexponents A_1_/(A_1_ + A_2_) and A_2_/(A_1_ + A_2_) for the iYTnC2 indicator, where A_1_ and A_2_ are pre-exponential factors in the association curve equation ΔFlu(t) = A_1_*exp.(-K_obs1_*t)-A_2_*exp.(-K_obs2_*t). **d** Calcium dissociation kinetics for the iYTnC2, NTnC and GCaMP6f GECIs. Starting concentration of Ca^2+^ was 1000 nM

Ca^2+^ association and dissociation kinetics were further studied for the iYTnC2 indicator with stopped-flow fluorimetry using NTnC and GCaMP6f GECIs as references. Association curves for iYTnC2 were bi-exponential in the range of 100–1300 nM Ca^2+^ concentrations (Fig. 2a). These two exponents corresponded to the observed fast and slow Ca^2+^ association rates constants k^on^_obs1_ and k^on^_obs2_, respectively (Fig. 2b, c). The relative contribution of the fast exponent was predominant (57–93%) for all Ca^2+^ concentrations tested (Fig. 2c), so all further calculations will correspond to the fast exponent. The minimum and medium calcium concentrations in the cytoplasm of active neurons range between 300 and 1300 nM Ca^2+^. At these calcium concentration ranges, iYTnC2 demonstrates a 5.6-fold (at 300 nM free Ca^2+^) to 2.5-fold (at 1300 nM free Ca^2+^) faster kinetics of Ca^2+^ binding than the GCaMP6f GECI. Fitting the dependence of observed Ca^2+^ association rate constants on Ca^2+^ concentrations to the equation k_obs_ = k_on_ × [Ca^2+^]^n^ + k_off_ allowed us to estimate Hill coefficient and the dissociation constant. Both K_d_^kin^ and Hill coefficient values for iYTnC2, NTnC, and GCaMP6f indicators were rather similar to those determined from the equilibrium studies (Table 1). The half-time of iYTnC2-Ca^2+^ dissociation was 5-fold less than the NTnC-Ca^2+^ complex and 1.7-fold larger than the commonly used indicator GCaMP6f with Ca^2+^ ions (Fig. 2d, and Table 1). Fast association-dissociation kinetics of the iYTnC2 indicator with calcium ions is advantageous for monitoring fast calcium activity in vivo.

### Calcium-dependent response of the iYTnC2 indicator in HeLa mammalian cells

**Fig. 3:**
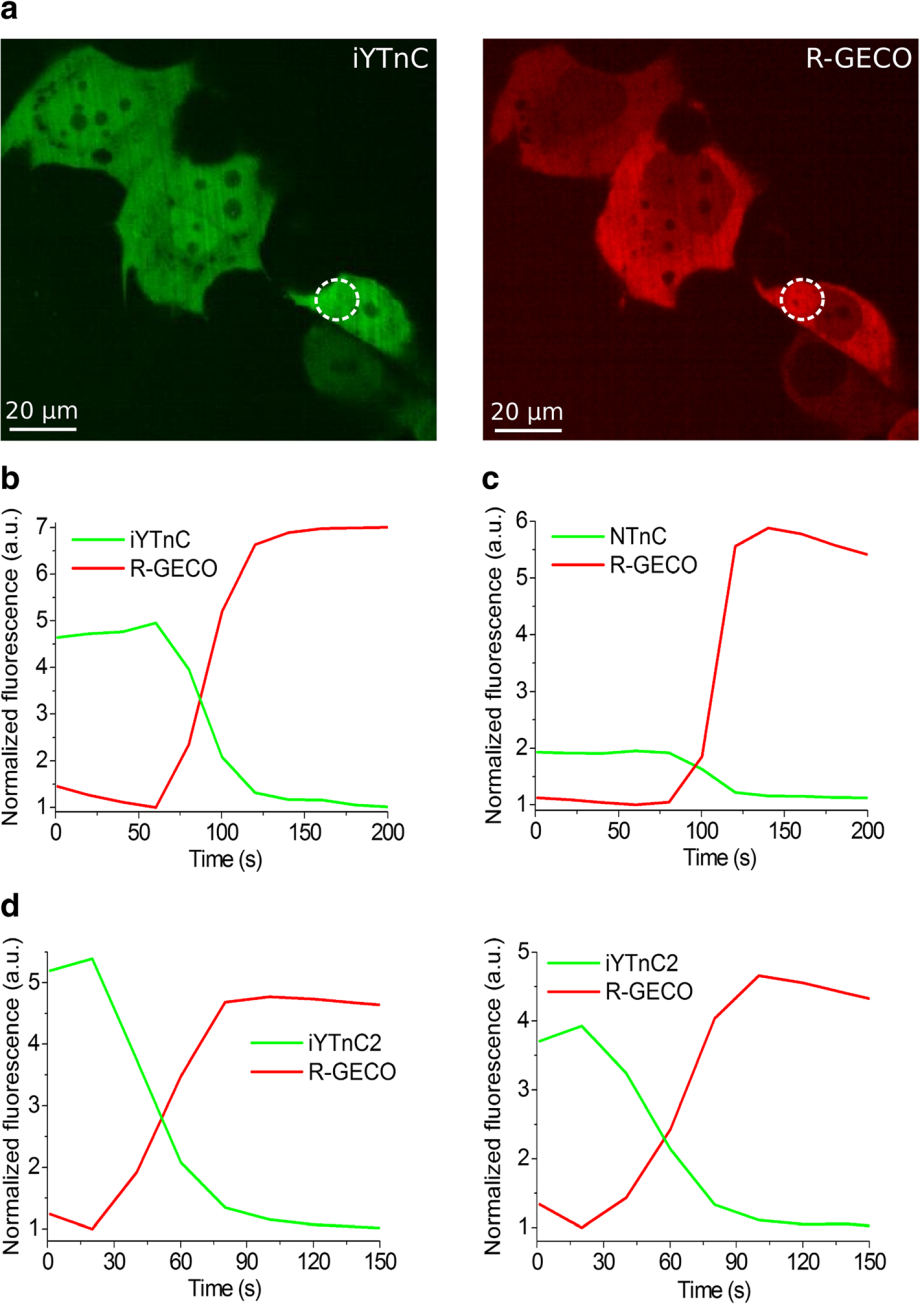
Response of the iYTnC and iYTnC2 indicators to Ca^2+^ concentration changes in HeLa cells. **a** Confocal images of HeLa Kyoto cells co-expressing green iYTnC (**a**, left) and red R-GECO1 (**a**, right) calcium indicators. **b**-**d** The graphs illustrate changes in green fluorescence of iYTnC (**b**), NTnC (**c**) or iYTnC2 (**d**) indicators and in red fluorescence of the reference co-expressed R-GECO1 GECI in response to addition of 2 mM CaCl_2_ and 2.5 μM ionomycin. The changes in panel **b** correspond to the area indicated with white circles in the panel **a**

To characterize the behavior of the iYTnC2 indicator in mammalian cells, we studied its response to the Ca^2+^ ions variations in the HeLa Kyoto cells. After the addition of 2 mM external CaCl_2_ with ionomycin, the indicator demonstrated a 4.2 ± 1.0-fold maximal change of its fluorescence in approximately 2 min, which is 2.7-fold larger than the relative response of NTnC and 1.3-fold less than the response of the spectrally distinct R-GECO1 GECI [12] co-expressed in the same cells (Fig. 3). The red calcium indicator R-GECO1 and iYTnC2 demonstrated similar dynamics (Fig. 3d). Hence, in the cytoplasm of mammalian cells, the iYTnC2 indicator demonstrates higher fluorescence contrasts in response to variations in Ca^2+^ concentration in comparison with NTnC GECI.

### Visualization of spontaneous neuronal activity in dissociated culture using iYTnC2 indicator

**Fig. 4:**
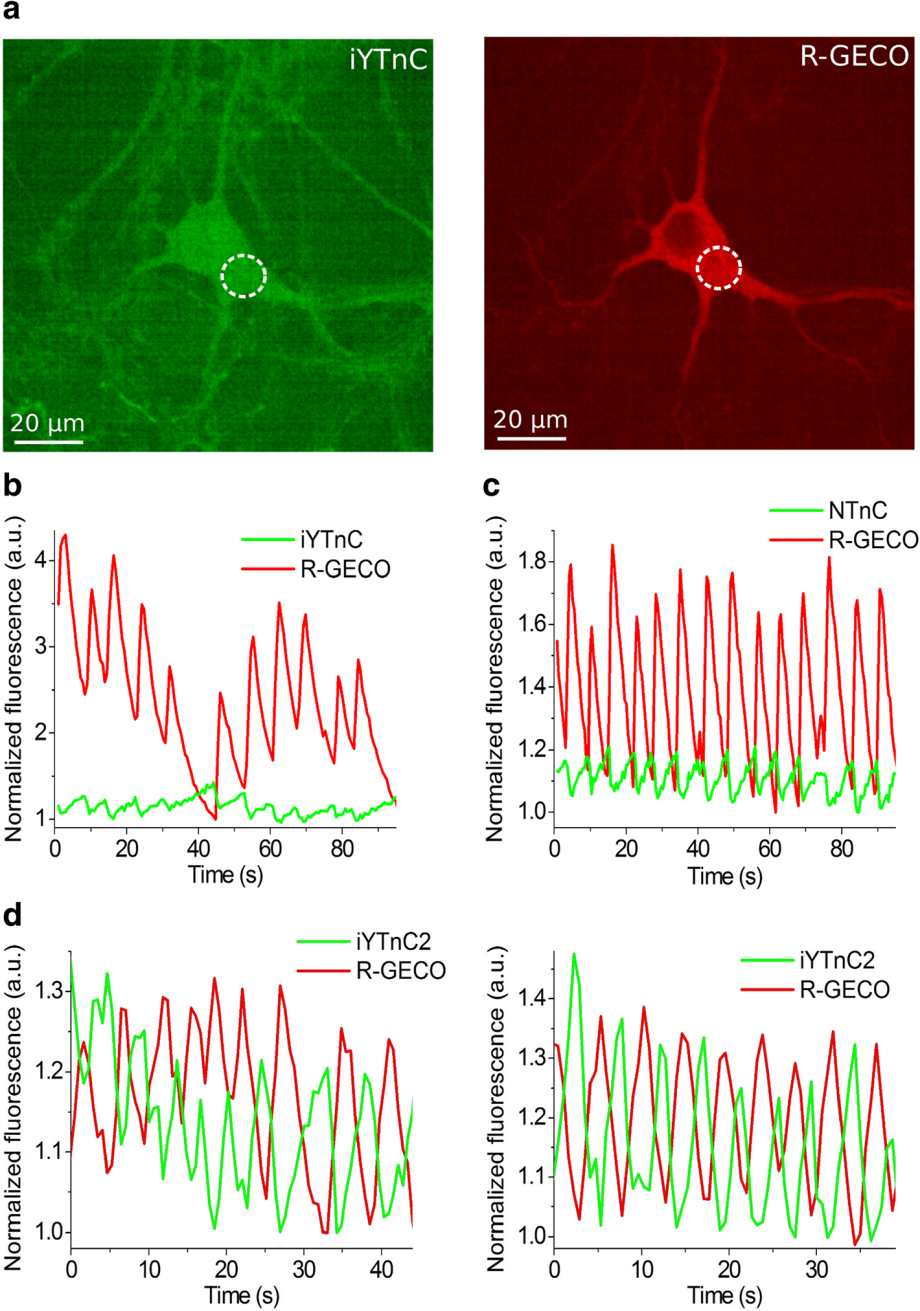
Response of iYTnC and iYTnC2 to Ca^2+^ variations as a result of spontaneous activity in cultured neurons. **a** Dissociated neuronal culture co-expressing iYTnC (**a**, left) and R-GECO1 (**a**, right) calcium indicators. **b** - **d** The graphs illustrate changes in red fluorescence of R-GECO1 (excitation 561 nm) and green fluorescence of iYTnC (**b**), NTnC (**c**) or iYTnC2 (**d**) (excitation 488 nm) as a result of spontaneous activity in neuronal culture. The graph on panel **b** illustrates changes in fluorescence in the area indicated with white circle in panel **a**. The minimal fluorescence values were normalized to the unit

To assess the functionality of the iYTnC2 indicator in neurons, we compared its response during spontaneous activity of dissociated neuronal cultures with those of NTnC and R-GECO1 GECIs. With this aim, we co-transduced neuronal cultures with recombinant AAVs of DJ serotype (rAAVs) particles encoding iYTnC2 or NTnC green indicators together with the reference R-GECO1 red calcium indicator under the control of CAG promoter. Spontaneous activity of neurons in two- to three-week-old cultures was accompanied with a decrease in green fluorescence of iYTnC2 and NTnC indicators with normalized ΔF/F values of 96% ± 31% and 22.7% ± 3.4%, respectively, relative to the red fluorescence of R-GECO1 (Fig. 4c, d). Hence, in neurons, iYTnC2 demonstrated a significant 4-fold larger normalized ΔF/F values as compared with those values for both parental iYTnC and NTnC indicators (Fig. 4b-d). The kinetics of iYTnC2 was practically identical to that of R-GECO1. The rise half-times for iYTnC2 and R-GECO1 expressing in the same neurons were practically the same, i.e. 0.8 ± 0.2 and 0.9 ± 0.3 s, respectively. The decay half-times for the iYTnC2 and R-GECO1 indicators were also similar, i.e. 1.1 ± 0.5 and 1.2 ± 0.3 s, respectively. Overall, these data indicate that the iYTnC2 indicator significantly outperformed both NTnC and iYTnC indicators in neurons.

### Characterization of iYTnC2 performance in neurons using whole-cell patch-clamp recording

**Fig. 5:**
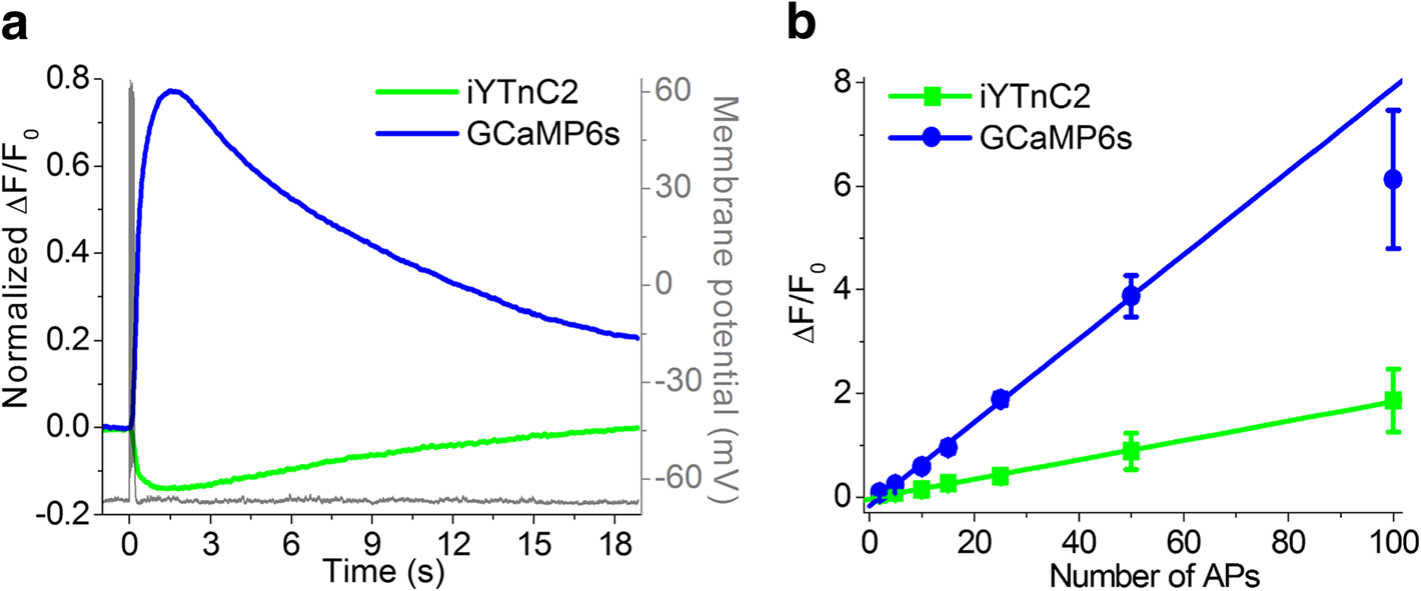
Fluorescence changes in GECI-expressing neurons in dissociated culture in response to intracellularly induced APs **a** Fluorescence changes in iYTnC2- and GCaMP6s- expressing cells to the train of 10 APs intracellularly induced with a frequency of 50 Hz. Ca^2+^ responses were averaged across representative recorded neurons in different wells (N = 6 for GCaMP6s and N = 10 for iYTnC2). Example of intracellular recording (grey) was taken from one representative cell. **b** Dependence of the amplitudes of responses induced by different numbers of APs in neurons expressing iYTnC2 and GCaMP6s. The linear regression was calculated for the 2–50 APs subset for both iYTnC2 and GCaMP6s. In the range of 2 to 50 APs the dependence is linear for both indicators while the amplitude of response to 100 APs in GCaMP6s-expressing neurons lies well below the linear regression line. At the same time response of iYTnC2 to 100APs is located directly on 2–50 regression line, i.e. dependence remains linear even for responses to strong stimulation. Values are shown as the means ± SEM

To characterize the performance of the iYTnC2 sensor in neurons, we compared fluorescence responses of cultured neurons expressing iYTnC2, GCaMP6s, and/or R-GECO1 GECIs via intracellular stimulation using whole-cell patch recording. Neurons of primary dissociated culture were co-transduced with iYTnC2 or GCaMP6s green indicators together with the reference R-GECO1 red GECI. In the first series of these experiments, we measured the fluorescent changes in neurons using either 470 or 530 nm excitation in response to the train of 10 action potentials (APs) induced intracellularly at a 50 Hz frequency. Responses of R-GECO1 to 10 APs were then used for normalization of iYTnC2 and GCaMP6s responses in the same cell. Non-normalized responses of neurons expressing iYTnC2 and R-GECO1 to 10APs are shown in Additional file 9: Figure S6. Altogether 9 cells in 4 wells for iYTnC2 and 7 cells in 3 wells for GCaMP6s from the same culture were recorded. Both indicators demonstrated fast and reliable changes in fluorescence levels in response to 10 APs train. As expected, intracellular stimulation of GCaMP6s-expressing neurons induced an abrupt increase in green fluorescence, while in response to the same stimulation iYTnC2-expressing cells showed a drop in fluorescence (Fig. 5a). Neurons expressing iYTnC2 showed faster kinetics of the Ca^2+^ responses as compared with GCaMP6s-positive cells. Thus, the half-rise time for iYTnC2 was 1.2-fold lesser than that for GCaMP6s (258 ± 35 ms and 304 ± 11 ms correspondingly; *p* < 0.05, Mann-Whitney Rank Sum Test; Additional file 10: Table S2). Half-decay times for both GECIs were practically identical. At the same time, signal-to-noise ratio (SNR) and ΔF/F were greater for Ca^2+^ responses measured in GCaMP6s-expressing neurons in comparison to iYTnC2-expressing cells (*p* < 0.01, Mann-Whitney Rank Sum Test).

We also estimated linearity of responses elicited by a different number of APs in the neurons expressing iYTnC2 and GCaMP6s GECIs using intracellular stimulation. We recorded responses to 2–100 APs induced via patch pipette using a 50 Hz stimulation frequency. In the range of 2 to 50 APs, both indicators showed a linear dependence of maximal ΔF/F values from the number of APs (Fig. 5b). However, the response of GCaMP6s to 100 APs showed notable saturation while responses of iYTnC2 indicator remained linear on the whole range tested (2–100 APs) (Fig. 5b). Thus, in neurons, iYTnC2 robustly and linearly responded to intracellular stimulations and demonstrated kinetics of association-dissociation with Ca^2+^ ions similar or even faster than that for the GCaMP6s indicator.

### Visualization of calcium transients in vivo in hippocampus of freely moving mice using nVista miniscope

**Fig. 6:**
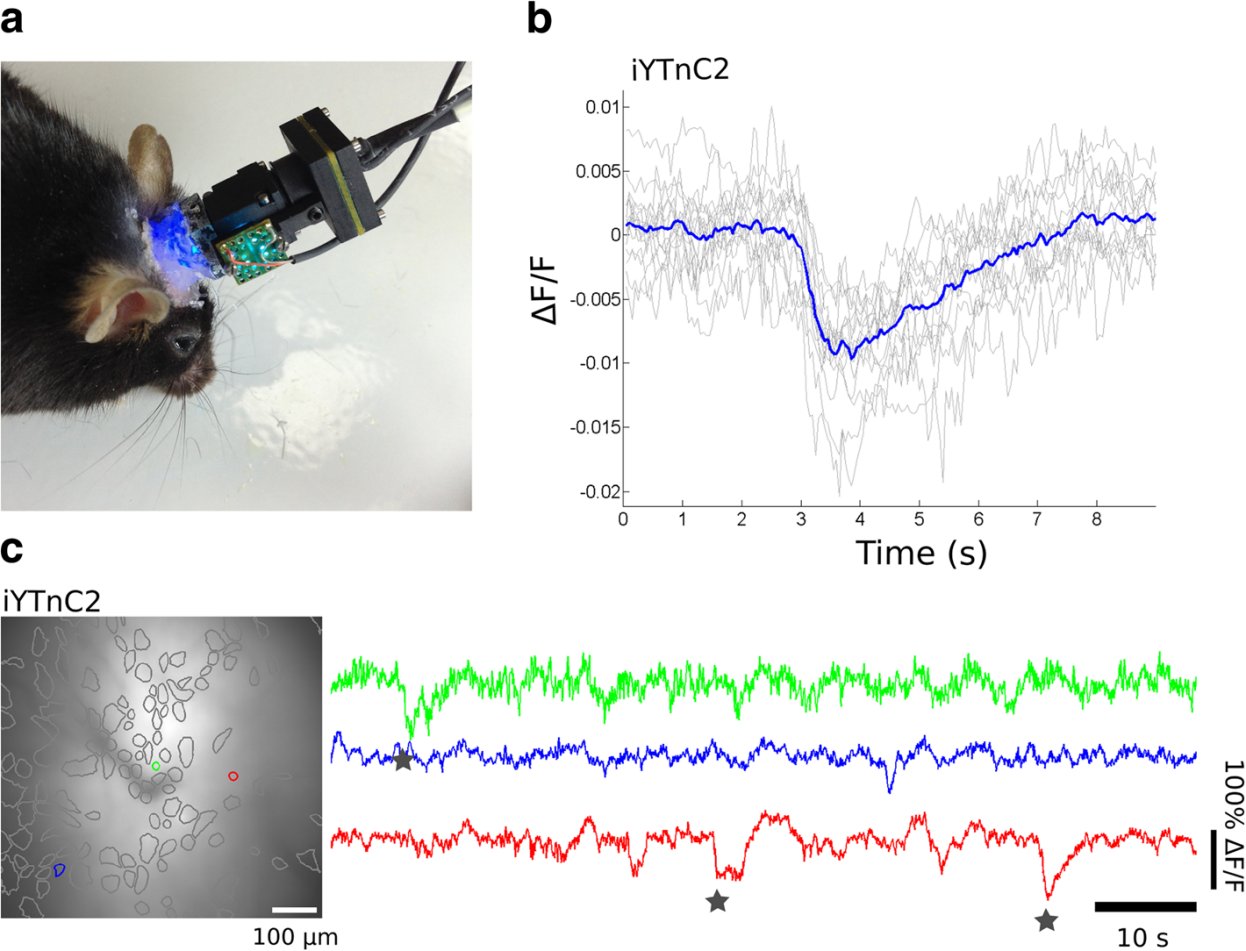
Spontaneous calcium activity of neurons in hippocampus of freely behaving mouse visualized with iYTnC2 and nVista HD system. **a** Photo of nVista HD miniature microscope head-mounted to the mouse. **b** Detected calcium spikes and the average one; spikes exceeding 4 MAD threshold were aligned at the moment of the very start of the peak (0 s). **c** Spatial filters and sample traces obtained from an imaging session with a freely behaving mouse expressing iYTnC2. Stars over traces denote spikes that were counted as 4 MAD threshold crossings. The sensor was delivered to the hippocampus by means of rAAV (AAV-*CAG*-NES-iYTnC2) particles

Finally, we applied iYTnC2 for visualization of neuronal calcium activity in vivo in the hippocampus of freely moving mice using miniaturized nVista microscope. This experiment was important to address whether the limited brightness found in vitro would restrict the utilization of iYTnC2 indicator for in vivo applications. To address this question, we used iYTnC2 to visualize neuronal calcium activity in the CA1 area of the hippocampus of freely moving mice during the exploration of an open field arena. We used an open field arena because these spatial tasks are commonly utilized in a number of behavioral paradigms. We installed the nVista microscope over the microendoscope lens implanted into the hippocampus of mice transduced with rAAV particles carrying iYTnC2 calcium indicator under the control of the CAG promoter. We captured calcium data at the frame rate of 20 fps and identified individual cells, some of which demonstrated temporal dynamics with inverted calcium spikes. The identified cells and examples of temporal dynamics and analysis of their calcium activity are presented in Fig. 6 and Additional file 11: Figure S7. As expected, the average calcium spike had rise and decay half-times of 0.7 ± 0.3 and 1.8 ± 0.9 s, respectively (Fig. 6b). Additionally, the averaged peak ΔF/F value and SNR value were estimated as 0.01 ± 0.002 and 6.5 ± 1.8, respectively. Thus, the iYTnC2 calcium indicator can be successfully used to visualize hippocampal neuronal dynamics in freely moving mice with nVista miniaturized microscope.

## Discussion

Using directed molecular evolution in bacteria, we developed a new genetically encoded green calcium indicator called iYTnC2 that has the NTnC-like design with enhanced fluorescence contrast and kinetics. The iYTnC2 indicator had 21 mutations relative to the original template (Additional file 3: Figure S2). Among these mutations, 14, 2 and 5 mutations were located in the fluorescent, sensory and linker parts, respectively. In the fluorescent domain, 5 mutations were internal and buried in the β-barrel, and 9 others were external. These external mutations may be necessary for efficient protein folding and interaction with the sensory unit. Internal mutations should act primarily to adjust the fluorescent properties of the chromophore. The four internal mutations F64L, K69Q, I240V, and Y276H, corresponded to the positions 64, 69, 167, and 203 in GFP, respectively (Additional file 3: Figure S2). According to the crystal structure of EGFP, they are located close to the chromophore [16]. The fifth internal mutation was found outside of the immediate surroundings of the chromophore and is unlikely to significantly affect chromophore fluorescence. Mutations V154E and C155S are located in the sensory part and may have an impact on Ca^2+^ binding or kinetics of the iYTnC2 indicator.

Compared with other commonly used indicators, iYTnC2’s length is ~ 100 amino acids shorter and has half the number of Ca^2+^-binding sites similar to the NTnC indicator (Additional file 1: Figure S1). The small molecular size iYTnC2 indicator is advantageous for packaging into the viral particles which have limited capacity [7]. The reduced number of Ca^2+^-binding sites should allow higher iYTnC2 indicator expression level in the neurons to get the same impact on endogenous free calcium concentration as compared with other GCaMP-like sensors which have four Ca^2+^-binding sites.

iYTnC2 has an inverted phenotype, i.e., its green fluorescence reduces upon the binding of Ca^2+^ ions similarly to the NTnC progenitor. This inverted fluorescent phenotype makes simple visualization of the resting neurons and cells with low concentration of free calcium and simplifies installation of nVista like systems for in vivo imaging. While the animal is under anesthesia the certain regions of the brain have the relatively quiet activity state of neurons and utilization of inverted iYTnC2 indicator ensures a lot of fluorescent cells for easy visualization in contrast to the un-inverted positive fluorescent GECIs. Hence, calcium indicators with the inverted phenotype provide additional reliable orienteers for the adjustment of the imaging volume during miniscopes installation.

We have characterized the main properties of the newly developed iYTnC2 indicator in vitro. The binding of iYTnC2 to Ca^2+^ ions is accompanied by a transition from one form of the chromophore (with absorbance at 499 nm) into another form (with absorbance at 410 nm). The 499- and 410-nm absorption bands with fluorescence maxima at 518 and 520 nm, respectively, can be attributed to the deprotonated, denoted as form A, and protonated, denoted as form B, forms of GFP-like chromophore, similar to those observed for GFP [17]. We assume that the high fluorescence contrast of the iYTnC2 indicator is due to the effective transition from the form A into the form B, the opposite to the NTnC indicator for which similar transition was less efficient [7]. In vitro, at a concentration of Mg^2+^ ions that resembles that in neurons, the iYTnC2 indicator shows a fluorescence contrast 2.8-fold higher than the NTnC indicator however it is still lower than the published in vitro contrasts for GCaMP6 and R-GECO1 indicators [12, 18]. iYTnC2 has 6-fold lower brightness and similar photostability as compared with NTnC in vitro. According to the stopped-flow experiments, iYTnC2 demonstrates Ca^2+^ dissociation kinetics 5-fold faster or 1.7-fold slower to that for NTnC and GCaMP6f, respectively. Depending on Ca^2+^ concentration in the range of 300–1300 nM, Ca^2+^ association kinetics of iYTnC2 is 5.6–2.5-fold faster than that for GCaMP6f. Faster kinetics makes iYTnC2 a perspective indicator for monitoring neuronal calcium activity in vivo.

We have expressed the iYTnC2 indicator and characterized its response in cultured mammalian and neuronal cells. iYTnC2 could reliably report variations in Ca^2+^ ion levels induced by ionomycin in mammalian cells, and, according to fluorescence contrast, iYTnC2 outperforms NTnC indicator by 2.7-fold. iYTnC2 demonstrated a 4-fold higher ΔF/F response in neuronal cultures as compared with iYTnC and NTnC GECIs. According to in vitro characteristics of iYTnC2 and iYTnC, the improved contrast of iYTnC2 in neurons could be explained by the reduced Mg^2+^-dependence of its fluorescence contrast and affinity to Ca^2+^ ions. Hence, the mutations V154E, C155S and G194D which are located in the sensory part reduced Mg^2+^-dependence of the contrast and affinity to Ca^2+^ ions of the iYTnC2 indicator.

Using whole-cell patch-clamp recording we demonstrated linear and robust response of iYTnC2 indicator to intracellular stimulations which induced 2–100 APs in neurons while GCaMP6s showed notable saturation at 100 APs. This saturation correlates with 2-fold higher calcium affinity of GCaMP6s as compared with iYTnC2 [18].

Finally, we successfully utilized iYTnC2 for in vivo visualization of neuronal dynamics in hippocampal brain area of mice. During installation of nVista miniscope, the inverted fluorescent phenotype of iYTnC2 indicator was useful for fine adjustments of the focal plane and field of view. The averaged peak ΔF/F value and SNR value for iYTnC2 were estimated as 0.01 ± 0.002 and 6.5 ± 1.8, respectively. These values were close to ΔF/F value of 0.012 ± 0.004 and SNR value of 4.4 ± 1.1 observed earlier for NTnC indicator in the similar in vivo application [7]. Thus, the iYTnC2 calcium indicator can successfully visualize neuronal transients in hippocampus of freely moving mice with nVista miniscope.

## Conclusions

In conclusion, we expect that further exploration of NTnC-like design, with the aim of enhancing its brightness and ΔF/F response in neurons, may result in indicators with performance levels similar or superior to those of GECIs with conventional designs.

## Additional files

Additional file 1: **Figure S1.** Schematic representation of FRET-based, cpFP-based, and NTnC-like indicator families in the Ca^**2**+^-bound state. **a** FPs are shown as cylinders, and tsTnC, CaM and M13-peptide are shown in dark grey, light grey, and speckled grey, respectively. **b** Schematic representation of the composition of original library and **c** schematic representation of the iYTnC indicator function. The EYFP fluorescent part is shown as intense or light green large cylinders before or after binding Ca^**2**+^ ions, respectively; tsTnC domains are shown as grey small tubes; Ca^**2**+^ ions are shown as dots. The Additional file 1: Figure S1 was adopted with modifications from ref. [7]. (TIFF 978 kb)

Additional file 2: Supplementary Methods. (PDF 194 kb)

Additional file 3: **Figure S2.** Alignment of the amino acid sequences for the original library, iYTnC, iYTnC2 and NTnC calcium indicators. Alignment numbering follows that of iYTnC. Residues from fluorescent part buried in β-can are highlighted with green. Residues that are forming chromophore are selected with asterisk. Mutations in iYTnC and iYTnC2 related to the initial library including linkers between fluorescent and indicator parts are highlighted with red. Residues that are forming Ca^2+^-binding loops are highlighted with blue. (PDF 13 kb)

Additional file 4: **Table S1.** In vitro properties of purified iYTnC compared to NTnC. (PDF 115 kb)

Additional file 5: **Figure S3.** In vitro properties of the purified iYTnC indicator. **a** Absorbance spectra for iYTnC in Ca^2+^-bound or Ca^2+^-free states at indicated pH values. **b** Excitation and emission spectra for iYTnC in Ca^2+^-free state at pH 7.2. **c** Fluorescence intensity for iYTnC in Ca^2+^-free and Ca^2+^-bound states and their dynamic range as a function of pH. Error represents the standard deviation for the average of three records. **d** Ca^2+^ titration curves for iYTnC and GCaMP6f in the absence and in the presence of 1 mM MgCl_2_. **e** Maturation curves for iYTnC, NTnC in Ca^2+^-free state, and mEGFP. **f** Photobleaching curves for iYTnC, NTnC in Ca^2+^-free state, and mEGFP. The power of light before objective lens was 7.3 mW/cm^2^. (TIFF 765 kb)

Additional file 6: **Figure S4.** Calcium association and dissociation kinetics for the iYTnC and GCaMP6f indicators studied using stopped-flow fluorimetry. **a** Calcium association kinetics curves for iYTnC. **b** Observed Ca^2+^ association rate constants determined from association curves for iYTnC and control GCaMP6f GECIs. For the iYTnC indicator, fast (green) and slow (grey) exponents are shown. **c** Relative contribution of monoexponents A_1_/(A_1_ + A_2_) and A_2_/(A_1_ + A_2_) for the iYTnC indicator, where A_1_ and A_2_ are pre-exponential factors in the association curve equation ΔFlu(t) = A_1_*exp.(-K_obs1_*t)-A_2_*exp.(-K_obs2_*t). **d** Calcium dissociation kinetics for the iYTnC, NTnC and GCaMP6f GECIs. Starting concentration of Ca^2+^ was 1000 nM. (TIFF 429 kb)

Additional file 7: Supplementary Results. (PDF 131 kb)

Additional file 8: **Figure S5.** Size-exclusion chromatography for iYTnC2 protein. Fast protein liquid chromatography of iYTnC2 in 40 mM Tris-HCl (pH 7.5), 200 mM NaCl buffer supplemented with 5 mM CaCl_2_. (TIFF 2054 kb)

Additional file 9: **Figure S6.** Fluorescence changes in cultured neurons co-expressing indicators iYTnC2 and R-GECO1 to the intracellularly induced train of 10 APs. Ca^**2**+^ responses were averaged across representative recorded neurons in different wells (N = 9 for R-GECO1 and N = 10 for iYTnC2). Example of intracellular recording (black, bottom) was taken from the one representative cell. (TIFF 213 kb)

Additional file 10: **Table S2.** Characteristics of calcium ions responses to intracellular stimulation with 10 APs in neurons expressing iYTnC2 and GCaMP6s sensors in dissociated neuronal culture. (PDF 12 kb)

Additional file 11: **Figure S7.** Spike detection scheme. *t_on* and *t_off* are rise and decay half-times. (TIFF 1370 kb)

## Abbreviations

FP: Fluorescent protein
GECI: Genetically encoded calcium indicator
