# Supplementary figures and images for: Genetically encoded calcium indicator with NTnC-like design and enhanced fluorescence contrast and kinetics
Source: BMC Biotechnol. 2018 Feb 13;18:10. doi: 10.1186/s12896-018-0417-2 (PMC5812234; doi:10.1186/s12896-018-0417-2)

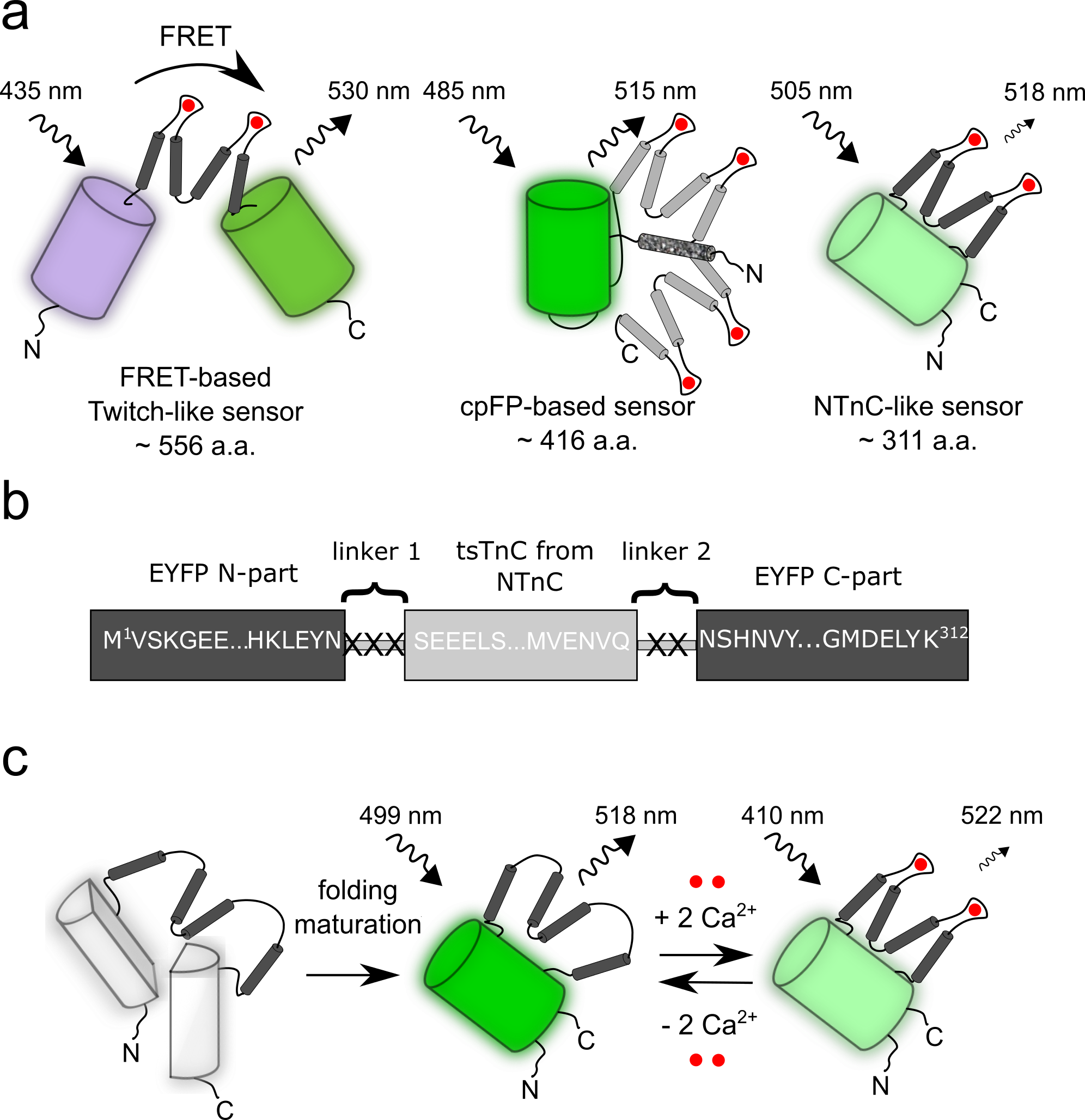

Supplement: Supplementary file 1 — Figure S1. Schematic representation of FRET-based, cpFP-based, and NTnC-like indicator families in the Ca2+-bound state. a FPs are shown as cylinders, and tsTnC, CaM and M13-peptide are shown in dark grey, light grey, and speckled grey, respectively. b Schematic representation of the composition of original library and c schematic representation of the iYTnC indicator function. The EYFP fluorescent part is shown as intense or light green large cylinders before or after binding Ca2+ ions, respectively; tsTnC domains are shown as grey small tubes; Ca2+ ions are shown as dots. The Additional file 1: Figure S1 was adopted with modifications from ref. [7]. (TIFF 978 kb) [file 12896_2018_417_MOESM1_ESM.tif]

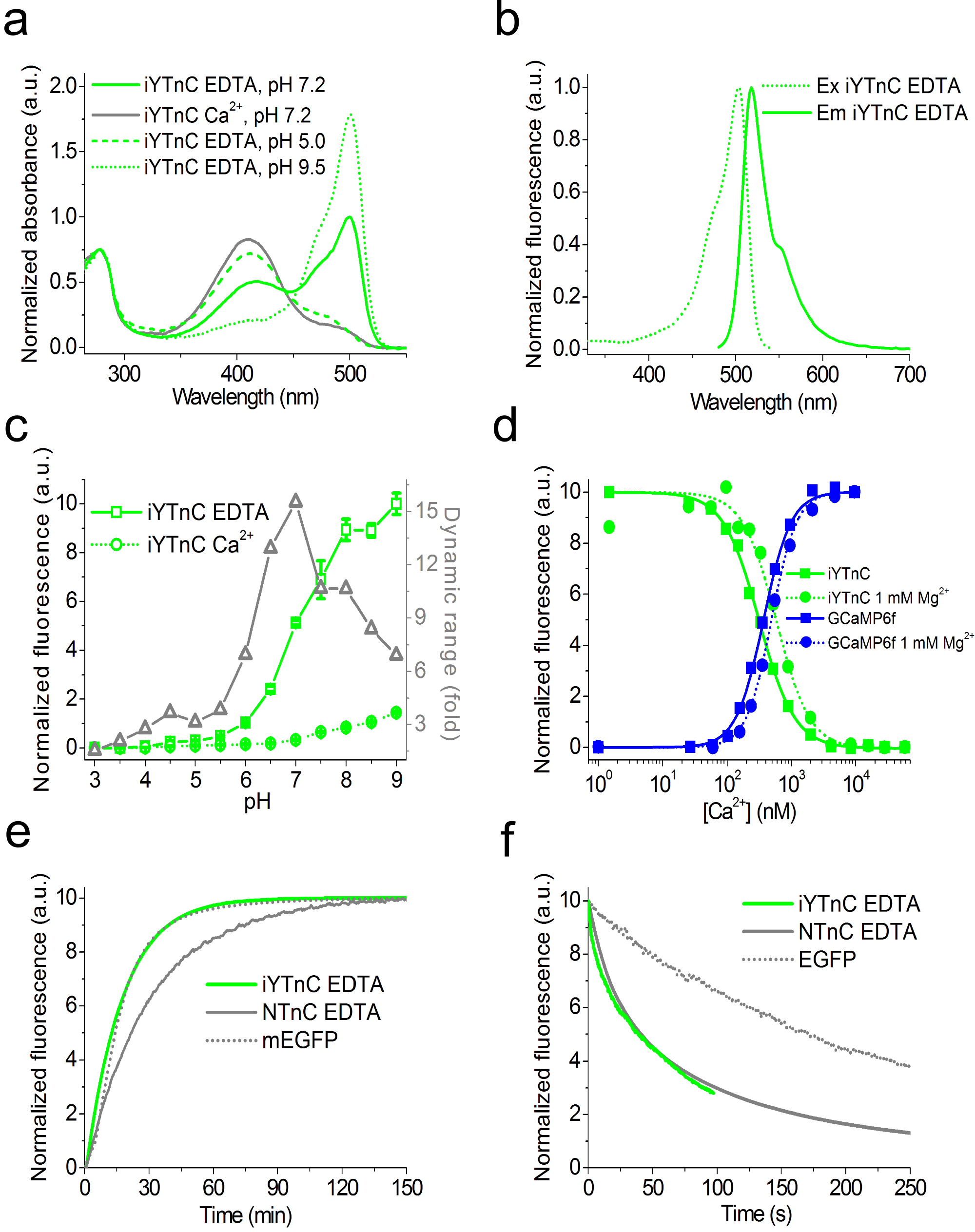

Supplement: Supplementary file 5 — Figure S3. In vitro properties of the purified iYTnC indicator. a Absorbance spectra for iYTnC in Ca2+-bound or Ca2+-free states at indicated pH values. b Excitation and emission spectra for iYTnC in Ca2+-free state at pH 7.2. c Fluorescence intensity for iYTnC in Ca2+-free and Ca2+-bound states and their dynamic range as a function of pH. Error represents the standard deviation for the average of three records. d Ca2+ titration curves for iYTnC and GCaMP6f in the absence and in the presence of 1 mM MgCl2. e Maturation curves for iYTnC, NTnC in Ca2+-free state, and mEGFP. f Photobleaching curves for iYTnC, NTnC in Ca2+-free state, and mEGFP. The power of light before objective lens was 7.3 mW/cm2. (TIFF 765 kb) [file 12896_2018_417_MOESM5_ESM.tif]

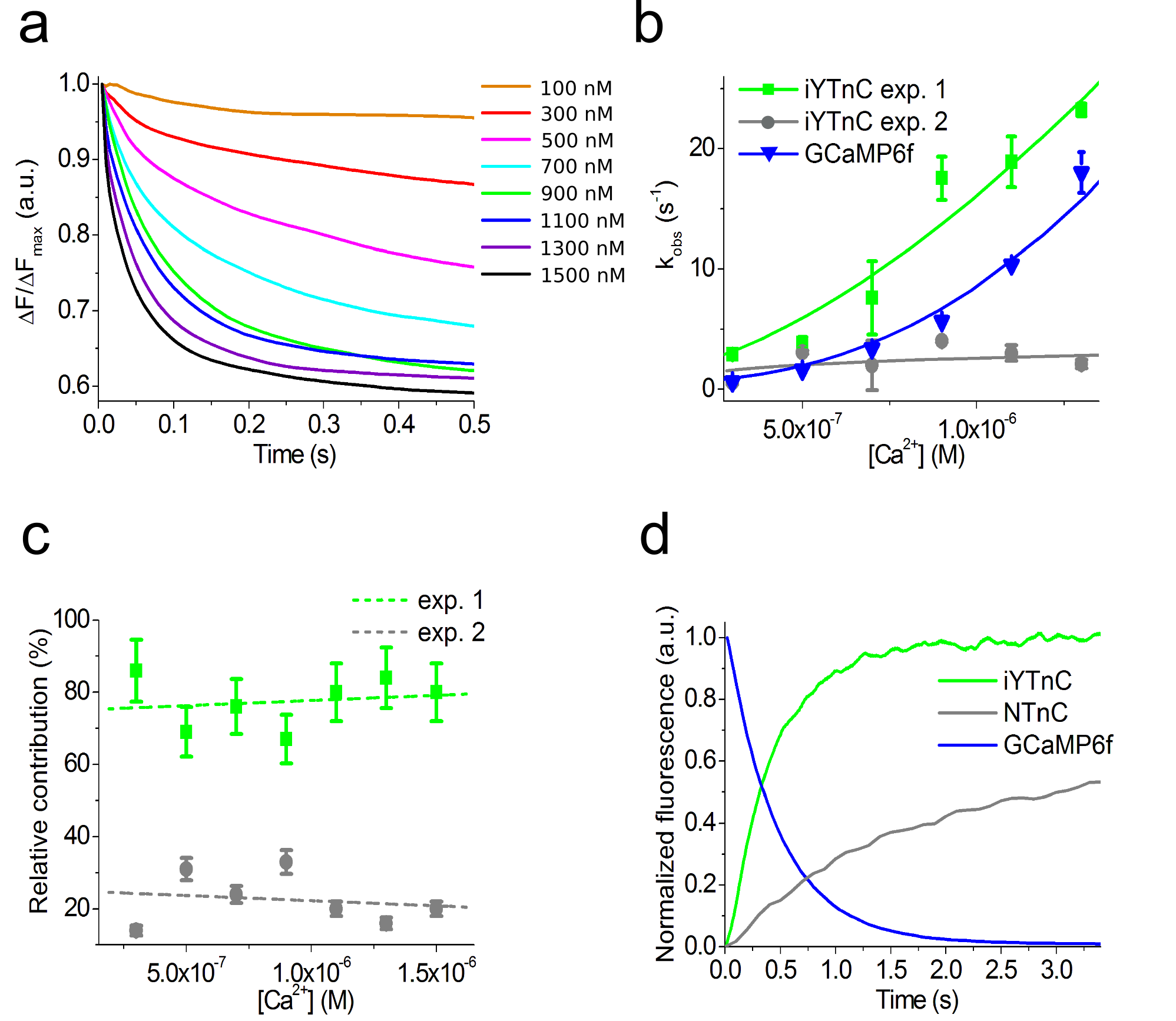

Supplement: Supplementary file 6 — Figure S4. Calcium association and dissociation kinetics for the iYTnC and GCaMP6f indicators studied using stopped-flow fluorimetry. a Calcium association kinetics curves for iYTnC. b Observed Ca2+ association rate constants determined from association curves for iYTnC and control GCaMP6f GECIs. For the iYTnC indicator, fast (green) and slow (grey) exponents are shown. c Relative contribution of monoexponents A1/(A1 + A2) and A2/(A1 + A2) for the iYTnC indicator, where A1 and A2 are pre-exponential factors in the association curve equation ΔFlu(t) = A1*exp.(-Kobs1*t)-A2*exp.(-Kobs2*t). d Calcium dissociation kinetics for the iYTnC, NTnC and GCaMP6f GECIs. Starting concentration of Ca2+ was 1000 nM. (TIFF 429 kb) [file 12896_2018_417_MOESM6_ESM.tif]

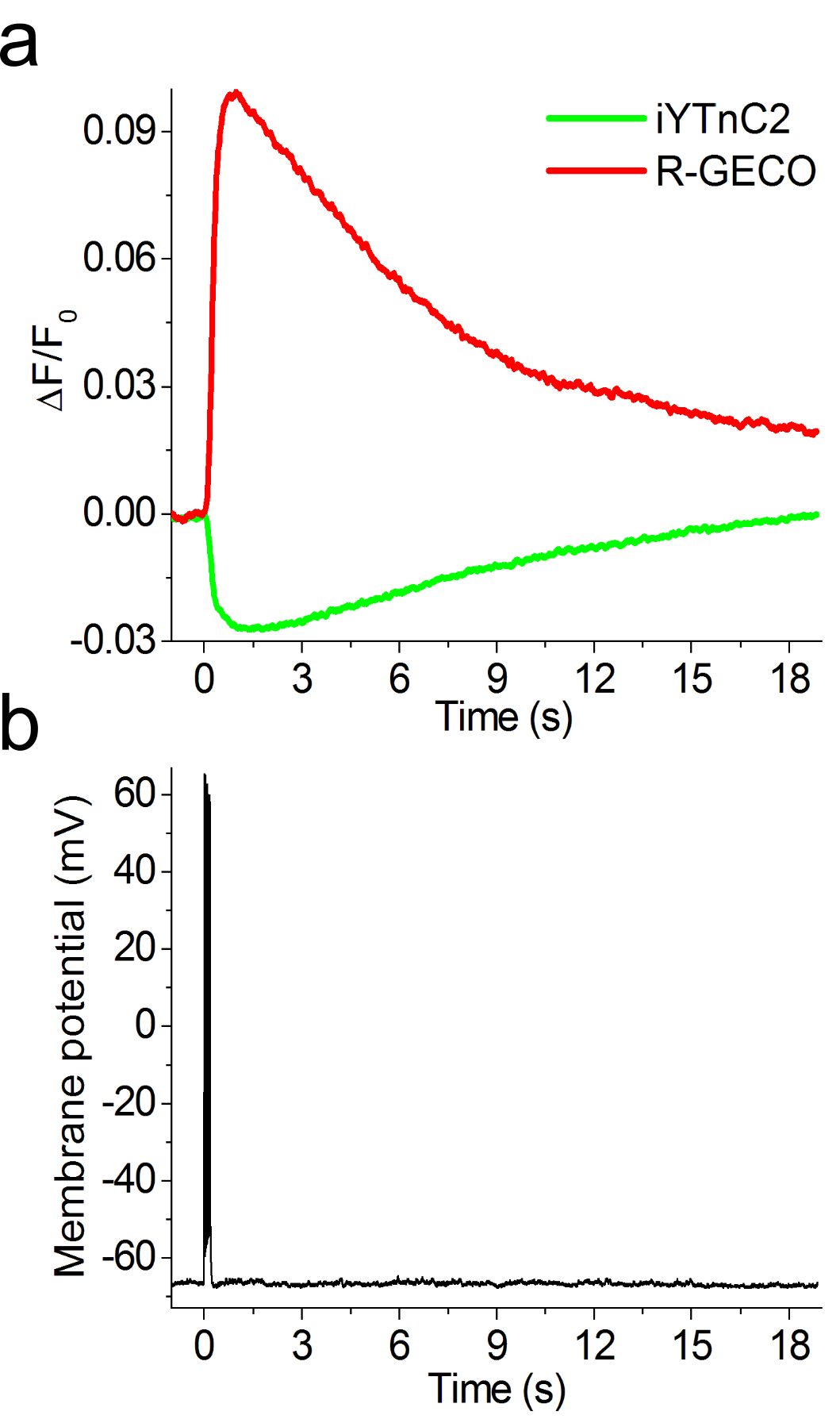

Supplement: Supplementary file 9 — Figure S6. Fluorescence changes in cultured neurons co-expressing indicators iYTnC2 and R-GECO1 to the intracellularly induced train of 10 APs. Ca2+ responses were averaged across representative recorded neurons in different wells (N = 9 for R-GECO1 and N = 10 for iYTnC2). Example of intracellular recording (black, bottom) was taken from the one representative cell. (TIFF 213 kb) [file 12896_2018_417_MOESM9_ESM.tif]

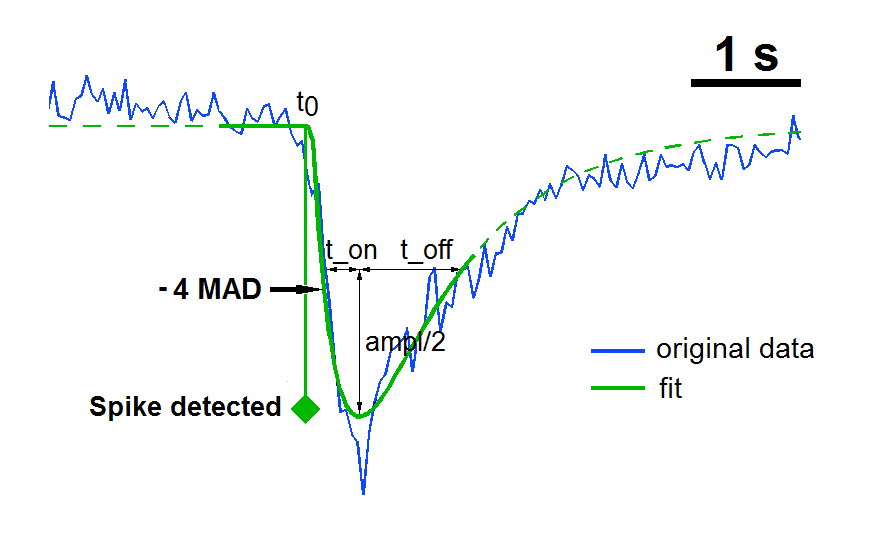

Supplement: Supplementary file 11 — Figure S7. Spike detection scheme. t_on and t_off are rise and decay half-times. (TIFF 1370 kb) [file 12896_2018_417_MOESM11_ESM.tif]
