# Supplementary material for: Genetically encoded calcium indicator with NTnC-like design and enhanced fluorescence contrast and kinetics
Source: BMC Biotechnol. 2018 Feb 13;18:10. doi: 10.1186/s12896-018-0417-2 (PMC5812234; doi:10.1186/s12896-018-0417-2)
Supplement: Supplementary file 2 — Supplementary Methods. (PDF 194 kb) [file 12896_2018_417_MOESM2_ESM.pdf]

Supplementary Methods

Image analysis with a NVista HD miniature microscope

All processing of calcium imaging data was made using the Mosaic software (Inscopix Inc.), and custom MATLAB scripts. First, all movies were spatially down-sampled by a factor of 2 in order to increase computation speed. Then, rigid body registration was made using a Mosaic routine based on TurboReg algorithm [1] to correct lateral displacements of the focal plane. After this, ΔF/F normalization was applied to the movies: $\Delta F/F = (F - F_0)/F_0$, where F_0 is intensity value for each pixel, averaged over time. For cell identification, spatial filters corresponding to individual cells were obtained using principal component analysis, followed by independent component analysis (PCA/ICA [2]). After a threshold of 50% of the filter's maximum intensity was applied to each filter, and all pixels with values lower than a threshold of one were set to zero. Filters with thresholds with low circularity, noisy appearance, and non-smooth borders were manually excluded from further analysis. After this, activity traces were extracted by applying threshold filters to ΔF/F movies. To correct neuropil contamination, for each trace the neuropil correction was made according to following equation: $F_{true}(t) = F(t) - F_{neuropil}(t)$, where F(t) is an extracted trace, and $F_{neuropil}(t)$ is a mean trace of all pixels inside 110 μm circle, with center at the brightest point of threshold filter; all threshold filters were excluded from this circle.

Calcium events (spikes) detection was performed whenever difference between a trace amplitude and its median value crossed the threshold of - 4 median absolute deviations (MADs, were calculated for each cell over the whole trace). The peak was approximated by function $f(t) = A(1 - e^{\frac{t-t_0}{\tau_{rise}}})e^{\frac{t-t_0}{\tau_{off}}}\theta(t-t_0) + B$, where t_0 is spiking time, t_{rise}, t_{off} - typical rise and decay times (not to be confounded with half-rise and half-decay times!), *A* – scale factor, *B* – background level and *θ* — Heaviside step function. To allow the detection of succeeding spikes after the given event, its fit was considered as a background for the next spike. Rise and decay half-times were measured as the times from the peak to half-peak on the left and right sides of the mean spike, respectively (see Fig. S7 in Additional file 11). Finally, signal-to-noise ratio (SNR) was quantified as peak ΔF/F response over 1 MAD.

Supplementary References

1. Thevenaz P, Ruttimann UE, Unser M: **A pyramid approach to subpixel registration based on intensity**. *IEEE Trans Image Process* 1998, **7**(1):27-41.
2. Mukamel EA, Nimmerjahn A, Schnitzer MJ: **Automated analysis of cellular signals from large-scale calcium imaging data**. *Neuron* 2009, **63**(6):747-760.
