# Supplementary material for: Genetically encoded calcium indicator with NTnC-like design and enhanced fluorescence contrast and kinetics
Source: BMC Biotechnol. 2018 Feb 13;18:10. doi: 10.1186/s12896-018-0417-2 (PMC5812234; doi:10.1186/s12896-018-0417-2)
Supplement: Supplementary file 3 — Figure S2. Alignment of the amino acid sequences for the original library, iYTnC, iYTnC2 and NTnC calcium indicators. Alignment numbering follows that of iYTnC. Residues from fluorescent part buried in β-can are highlighted with green. Residues that are forming chromophore are selected with asterisk. Mutations in iYTnC and iYTnC2 related to the initial library including linkers between fluorescent and indicator parts are highlighted with red. Residues that are forming Ca2+-binding loops are highlighted with blue. (PDF 13 kb) [file 12896_2018_417_MOESM3_ESM.pdf]

```
             -1         10        20        30        40        50
              |          |         |         |         |         |
Library       MVSKGEELFTGVVPILVELDGDVNGHKFSVSGEGEGDATYGKLTLKFICTTGKLPVPW
iYTnC         MVSKGEELFTGVVPILVELVGDVNGHKFSVSGEGEGDATYGKLTLKFICTTGKLPVPW
iYTnC2        MVSKGEELFTGVVPILVELVGDVNGHKFSVSGEGEGDATYGKLTLKFICTTGKLPVPW
NTnC        MVSKGEEDNMASLPATHELHIFGSINGVDFDMVGQGSGNPNVGYEELNLKSTKGDLQFSP

             60        70        80        90       100       110
              |    *** |         |         |         |         |
Library     PTLVTTFGYGLKCFARYPDHMKQHDFFKSAMPEGYVQERTIFFKDDGNYKTRAEVKFEGD
iYTnC       PTLVTTLGYGLQCFARYPDHMKQHDFFKSAMPEGYVQERAIFFEDDGNYKTRAEVKFEGD
iYTnC2      PTLVTTLGYGLQCFARYPDHMKQHDFFKSAMPEGYVQERAIFFEDDGNYKTRAEVKFEGD
NTnC        WILVPHIGYGFHQYLPYPDGMS-PFQAAMVDGSGYQVHRTVQFEDGASLTVNYRYTYEGS

            120       130       140       150       160       170
              |         |         |         |         |         |
Library     TLVNRIELKGIDFKEDGNILGHKLEYNXXXSEEELSVCFRTFDKDGDGFIDREEFGGIIR
iYTnC       TLVNRIELKGIDFKEDGNILGHKLEYNLVRSEEELSEYFRTFDKDGDGFIDREEFGGIIR
iYTnC2      TLVNRIELKGIDFKEDGNILGHKLEYNLVRSEEELSESFRTFDKDGDGFIDREEFGGIIR
NTnC        HIKGEAQVKGTGFPADGPVMANSLT-AMVPSEEELSECFRTFDKDGDGFIDREEFGGIIR

            180       190       200       210         220       230
              |         |         |         |           |         |
Library     LTGEQLTDEDPDEIFGDSDTDKNGRIDFDEFLKMVENVQXX--NSHNVYIMADKQKNGIK
iYTnC       LTGEQLTDEDPDEIFGGSDTDKNGRIDFDEFLKMVENVQMF--NSHNVYIMADKQRNGIK
iYTnC2      LTGEQLTDEDPDEIFGDSDTDKNGRIDFDEFLKMVENVQMF--NSHNVYIMADKQRNGIK
NTnC        LTGEQLTDEDPDEIFGDSDTDKNGRIDFDEFLKMVENVQLSMADWCRSKMACP-NDKTLI

              240       250       260          270       280       290
                |         |         |            |         |         |
Library     VNFKIRHNIEDGSVQLADHYQQNTPIGDG---PVLLPDNHYLSYQSALSKDPNEKRDHMV
iYTnC       AHFKVCHNIEDGSVQLADHYQQNTPIGDG---PVLLPDNHYLSHRSALSKDPNEKRDHMV
iYTnC2      AHFKVCHNIEDGSVQLADHYQQNTPIGDG---PVLLPDNHYLSHRSALSKDPNEKRDHMV
NTnC        STLKWSYTTGNGKRYRSTARTTYTFAKPMAANYLKNQP-MYVFRKTELKHS----KTEMD

                 300       310
                   |         |
Library     LLEFVTAAGITLGMDELYK
iYTnC       LLESVTAVGITLGMDELYK
iYTnC2      LLESVTAVGITLGMDELYK
NTnC        FKEWQKAFTDVMGMDELYK
```
