# Supplementary material for: Genetically encoded calcium indicator with NTnC-like design and enhanced fluorescence contrast and kinetics
Source: BMC Biotechnol. 2018 Feb 13;18:10. doi: 10.1186/s12896-018-0417-2 (PMC5812234; doi:10.1186/s12896-018-0417-2)
Supplement: Supplementary file 4 — Table S1. In vitro properties of purified iYTnC compared to NTnC. (PDF 115 kb) [file 12896_2018_417_MOESM4_ESM.pdf]

Properties		Proteins			
		iYTnC		NTnC	
		apo	sat	apo	sat
**Absorbance maximum (nm)**		499 (416)	410	505	
**Emission maximum (nm)**		518 (516)	522	518	
**Quantum yield** [a]		0.43±0.04 (0.060±0.001)	0.04±0.03	0.71±0.05	0.65±0.04
**ε (mM^{-1} cm^{-1})**		36±2 (18.2±0.7) [c]	30±1 [b]	108±6 [b]	52±1 [c]
**Brightness (%)**		20 (1.4)	1.6	100	44
**Fluorescence contrast (fold)**	**0 mM Mg^{2+}**	14±1		2.0±0.3	
	**1 mM Mg^{2+}**	4.1±0.2		2.0±0.7	
**pKa**		7.0±0.1	7.7±0.1	6.09±0.07	6.08±0.02
**K_d (nM)** [d]	**0 mM Mg^{2+}**	315±8 [n=1.6±0.1]		84±6 [n=1.9±0.1]	
	**1 mM Mg^{2+}**	583±57 [n=1.5±0.1]		192±40 [2.0±0.4]	
**K_d^{kin} (nM)** [e]		300±200 [n=1.6±0.1]		94±9 [n=2.3±0.1]	
**k_{on} (s^{-1}× M^{-n})** [f]		$7±2 \times 10^{10}$		6×10^{15}	
**k_{off} (s^{-1})** [g]		2.4±0.1		0.8±0.1; 0.05±0.01 [h]	
**$t_{1/2}^{off}$ (s)** [g]		0.31±0.01		3.00±0.05	
**Maturation half-time (min)** [i]		13	ND	23	28
**Photobleaching half-time (s)** [j]		49±16	ND	40±8	70±5

[a] QYs were determined at pH 7.20. GCaMP6s in the saturated state (QY=0.61 [1]) and mTagBFP2 (QY=0.64 [2]) were used as reference standards for 499- to 505- and dim fluorescent 410- to 416-nm absorbing states, respectively.

[b] Extinction coefficients were determined by alkaline denaturation.

[c] Extinction coefficients were estimated relative to $iYTnC_{sat}$ or $NTnC_{apo}$ with the same concentration determined according to the absorbance at 280 nm.

[d] Hill coefficients are shown in square brackets. In the absence and the presence of 1mM Mg^{2+} ions, GCaMP6f control GECI had K_d value of 370±8 [2.03±0.08] and 492±10 [2.23±0.09] nM, respectively.

[e] K_d^{kin}, Hill coefficients and k_{on} values were obtained via fitting the observed association rates to the equation $k_{obs} = k_{on} \times [Ca^{2+}]^n + k_{off}$ (Fig. 2a). $K_d^{kinetic} = (k_{off}/k_{on})^{1/n}$. Hill coefficients are shown in square brackets. GCaMP6f has K_d^{kin} value of 450±300 nM [2.4±0.1].

[f] GCaMP6f has k_{on} value of $(3.5±2)\times 10^{15}$ $s^{-1}\times M^{-n}$.

[g] Refined k_{off} and $t_{1/2}^{off}$ values were determined from the dissociation kinetics records (Fig. 2d). GCaMP6f has $t_{1/2}^{off}$ and k_{off} values of 0.35±0.02 s and 2.109±0.002 s^{-1}, respectively.

[h] Unlike iYTnC kinetics, NTnC kinetics do not agree with the two-state model. NTnC kinetic curves were fitted to double exponentials. k_{off} values were estimated from double exponential decay with individual exponent contributions of 0.48:0.52.

[i] EGFP had a maturation half-time of 14 min. [j] mEGFP had a photobleaching half-time of 170±20 s.

References:

1. Chen TW, Wardill TJ, Sun Y, Pulver SR, Renninger SL, Baohan A, Schreiter ER, Kerr RA, Orger MB, Jayaraman V *et al*: **Ultrasensitive fluorescent proteins for imaging neuronal activity**. *Nature* 2013, **499**(7458):295-300.
2. Subach OM, Cranfill PJ, Davidson MW, Verkhusha VV: **An enhanced monomeric blue fluorescent protein with the high chemical stability of the chromophore**. *PloS one* 2011, **6**(12):e28674.
