# Supplementary material for: Genetically encoded calcium indicator with NTnC-like design and enhanced fluorescence contrast and kinetics
Source: BMC Biotechnol. 2018 Feb 13;18:10. doi: 10.1186/s12896-018-0417-2 (PMC5812234; doi:10.1186/s12896-018-0417-2)
Supplement: Supplementary file 7 — Supplementary Results. (PDF 131 kb) [file 12896_2018_417_MOESM7_ESM.pdf]

Supplementary Results

In vitro characterization of purified iYTnC

The *in vitro* characteristics of the purified iYTnC calcium indicator are summarized in Table S1 in Additional file 4. At pH 7.2 in the Ca^{2+}-free state $iYTnC_{apo}$ exhibited green fluorescence with emission peaks at 516 and 518 nm when excited at absorbance peaks at 416 and 499 nm, correspondingly. At pH 9.5, when $iYTnC_{apo}$ is in the deprotonated anionic fluorescent state, only one emission and one absorbance peaks were observed at 499 and 518 nm, respectively. At pH 5, when the chromophore of $iYTnC_{apo}$ protein is protonated, one emission and one absorbance peak were registered at 416 and 516 nm, respectively. In Ca^{2+}-saturated state $iYTnC_{sat}$ maximally absorbed at 410 nm and had dim fluorescence peaked at 522 nm (Fig. S3a, b in Additional file 5). In both the Ca^{2+}-free and Ca^{2+}-saturated states, the brightness of the 410-416-nm absorbing forms of iYTnC were 12-14-fold lower than that of the main fluorescent 499-nm absorbing form. The brightness of the fluorescent 499-nm absorbing form of $iYTnC_{apo}$ indicator in the Ca^{2+}-free state was 5-fold lower than that for the control NTnC GECI (Table S1 in Additional file 4). The maximal fluorescence contrast of 14±1-fold (through the text the reported error correspond to SD) for the iYTnC indicator between Ca^{2+}-free and Ca^{2+}-saturated states was in 6.7-fold larger than the contrast for the NTnC GECI.

First, we attempted to understand the factors that may contribute to the contrast of the iYTnC indicator. The iYTnC binding to calcium ions was accompanied by a 13.5-fold reduction in absorbance at 499 nm and increase in absorbance for the sensor at 410 nm. Absorbance contrast was the same as compared with the 13.5-fold fluorescence response (Table S1 in Additional file 4). Hence, binding of iYTnC to Ca^{2+} ions is accompanied by a transition from one form of the chromophore (with absorbance at 499 nm) into another form (with absorbance at 410 nm). The 499- and 410-nm absorbing forms with fluorescence maxima at 518 and 522 nm, respectively, can be attributed to the deprotonated, denoted as form A, and protonated, denoted as form B, GFP-like chromophore, similar to those observed for GFP [1]. Hence, the high fluorescence contrast of the iYTnC indicator is ensured by the effective transition from the form A into the form B in opposite to the NTnC indicator for which similar transition was incomplete [2].

Next, we characterized the dependence of iYTnC GECI fluorescence and contrast from pH. The iYTnC exhibited shift in p*K*a values from 7.0 in the Ca^{2+}-free state to 7.7 in the Ca^{2+}-bound state (Fig. S3c in Additional file 5, Table S1 in Additional file 4). As a result, the fluorescence of both states, as well as fluorescence contrast of iYTnC, showed dependence on pH within the physiological range of pH 7-8. Similarly to iYTnC, the commonly used GCaMP6s indicator based on pH-sensitive EGFP protein had also a pronounced pH-dependence of its $\Delta F/F$ response within the

pH range of 5-8 [2]. In contrast, NTnC indicator based on pH-stable mNeonGreen FP had lower sensitivity to pH changes [2, 3]. Hence, sensitivity to pH was probably inherited by iYTnC and NTnC GECIs from their fluorescent progenitors.

We further assessed the affinity of the iYTnC indicator to Ca^{2+} ions. According to equilibrium binding titrations experiments, iYTnC demonstrated an equilibrium K_d value of 315±8 nM which was 3.8-fold lower and practically similar to those values for NTnC and GCaMP6f, respectively (Fig. S3d in Additional file 5 and Table S1 in Additional file 4). Its affinity to Ca^{2+} ions was 1.3-2.1-fold weaker than those for the FRET indicators Twitch-1/2/3, which were based on the same TnC domain [4]. Probably, mutations in the linkers and sensory part decreased the affinity of the iYTnC sensor to Ca^{2+} ions. Equilibrium Hill coefficient for the iYTnC indicator was lower than those for NTnC and GCaMP6f GECIs evidencing decreased cooperativity of Ca^{2+} binding by iYTnC.

We next characterized the response of iYTnC to Ca^{2+} ions in the conditions that are similar to those in the cytoplasm of neurons. In the presence of 1 mM Mg^{2+}, purified iYTnC and NTnC demonstrated K_d values of 583±57 and 192±40 nM, respectively, which were in 1.9- and 2.3-fold larger than K_d values in the absence of Mg^{2+} ions (Fig. S3d in Additional file 5 and Table S1 in Additional file 4). At 1 mM Mg^{2+}, a concentration that resembles that in neuronal cytoplasm, fluorescence contrast of iYTnC dropped to the value of 4.1±0.2 which was 3.3-fold lower than in the absence of Mg^{2+} ions, but it was still in 2-fold higher than the contrast of NTnC indicator in the same conditions.

We also charaterized the maturation rate and photostability of iYTnC using NTnC and EGFP as the controls. At 37°C, the iYTnC indicator in the Ca^{2+}-free state matured till 50% for the 13 min and this process was 1.8-fold faster than for the NTnC and iYTnC's half-time was similar to the maturation half-time of 14 min for the EGFP (Fig. S3e in Additional file 5). Under a wide-field microscope equipped with a metal halide lamp, both the iYTnC and NTnC indicators in the Ca^{2+}-free state photobleached 4.5-fold faster than EGFP (Fig. S3f in Additional file 5).

Overall, iYTnC demonstrated up to 6.7-fold higher fluorescence contrast, 5-fold dimmer brightness, lower pH stability, 3.8-fold decreased affinity to Ca^{2+} ions, faster maturation rate and similar photostability as compared with NTnC *in vitro*.

Characterization of iYTnC calcium indicator kinetics using stopped-flow fluorimetry

Ca^{2+} association and dissociation kinetics were further studied for the iYTnC indicator with stopped-flow fluorimetry using NTnC and GCaMP6f GECIs as references. Association curves for iYTnC were bi-exponential in the range of 100-1300 nM Ca^{2+} concentrations (Fig. S4a in Additional file 6). These two exponents corresponded to the fast and slow observed Ca^{2+} association rates constants $k^{on}_{obs}1$ and $k^{on}_{obs}2$, respectively (Fig. S4b, c in Additional file 6). The relative contribution of the fast exponent was predominant (67-87%) for all Ca^{2+} concentrations tested (Fig. S4c in

Additional file 6) so all further calculations will correspond to the fast exponent. In the range of 300-1300 nM Ca^{2+} concentrations corresponding to the minimal and maximal elevated calcium concentrations in the cytoplasm of active neurons, iYTnC demonstrated 4.6-fold (at 300 nM free Ca^{2+}) to 1.3-fold (at 1300 nM free Ca^{2+}) faster kinetics of Ca^{2+} binding than GCaMP6f GECI. Fitting the dependence of observed Ca^{2+} association rate constants on Ca^{2+} concentrations to the equation $k_{obs} = k_{on} \times [Ca^{2+}]^n + k_{off}$ allowed us to estimate Hill coefficient and the dissociation constant. Both K_d^{kin} and Hill coefficient values for iYTnC, NTnC and GCaMP6f indicators were rather similar to those determined from the equilibrium studies (Table S1 in Additional file 4).The half-time of iYTnC-Ca^{2+} dissociation was 10-fold less than that for the NTnC-Ca^{2+} complex and similar to the complex of commonly used indicator GCaMP6f with Ca^{2+} ions (Fig. S4d in Additional file 6, and Table S1 in Additional file 4). Fast association-dissociation kinetics of the iYTnC indicator with calcium ions is advantageous for monitoring fast calcium activity *in vivo*.

Analysis of the fast kinetics of association-dissociation for the iYTnC indicator with Ca^{2+} ions allowed us to assume one of the possible mechanisms for this interaction. As we mentioned above, the association curves were bi-exponential (Fig. S4a in Additional file 6) so we could calculate the fast and slow observed Ca^{2+} association rate constants $k^{on}_{obs}1$ and $k^{on}_{obs}2$, respectively (Fig. S4b, c in Additional file 6). The first observed rate constant $k^{on}_{obs}1$ enlarged with increasing of Ca^{2+} concentration and hence it might characterize the process of binding Ca^{2+} ions to the iYTnC indicator. The second (slower) observed rate constant $k^{on}_{obs}2$ did not depend on concentration of Ca^{2+} ions (Fig. S4b in Additional file 6), so we suggested that this rate constant corresponded to the slow process that did not relate with interaction between the iYTnC indicator and Ca^{2+} ions but it rather reflected conformational changes of iYTnC protein that followed Ca^{2+} ions binding. Overall, our data evidence that calcium ions binding is a fast process (at $[Ca^{2+}] > 300$ nM) with moderate positive cooperativity (Hill coefficient of 1.6), and the slow step is the following conformational changes within the iYTnC complex with Ca^{2+} ions.

Calcium-dependent response of the iYTnC indicator in HeLa Kyoto mammalian cells

To characterize the behavior of the iYTnC indicator in mammalian cells, we studied its response to the Ca^{2+} ions variations in the HeLa Kyoto cells. After the addition of 2 mM external $CaCl_2$ with ionomycin, the indicator demonstrated a 4.2±0.8-fold maximal change of its fluorescence in approximately 2 min, which is 2.2-fold larger than the relative response of NTnC and 1.5-fold less than the response of R-GECO1 (Fig. 3a-c). When co-expressed in the same cells, the red calcium indicator R-GECO1 and iYTnC demonstrated similar dynamics (Fig. 3b). Hence, in the cytoplasm of mammalian cells, the iYTnC indicator demonstrates higher fluorescence contrasts in response to variations in Ca^{2+} concentration in comparison with NTnC GECI.

Visualization of spontaneous neuronal activity in dissociated culture using iYTnC indicator

To assess the functionality of the iYTnC indicator in neurons, we compared its response during spontaneous activity of dissociated neuronal cultures with those of NTnC and R-GECO1 GECIs. With this aim, we co-transduced neuronal cultures with recombinant AAVs (rAAVs) particles encoding iYTnC or NTnC green indicators together with reference R-GECO1 red calcium indicator under the control of CAG promoter. Spontaneous activity of neurons in two- to three-week-old cultures was accompanied by a decrease in green fluorescence of iYTnC and NTnC indicators with normalized $\Delta F/F$ values of 22.7±3.4% and 22.6±3.5%, respectively, relative to the red fluorescence of R-GECO1 (Fig. 4a-c). The kinetics of iYTnC was practically identical to that of R-GECO1. The rise half-times for iYTnC and R-GECO1 expressing in the same neurons were practically the same, i.e. 0.8±0.3 and 1.0±0.3 s, respectively. The decay half-times for the iYTnC and R-GECO1 indicators were also similar, i.e. 2.6±0.3 and 2.5±0.5 s, respectively. Overall, these data indicate that the iYTnC indicator monitors the spontaneous activity of neurons with similar or lower response as compared with NTnC and R-GECO1 indicators.

Supplementary References

1. Palm GJ, Zdanov A, Gaitanaris GA, Stauber R, Pavlakis GN, Wlodawer A: **The structural basis for spectral variations in green fluorescent protein**. *Nature structural biology* 1997, **4**(5):361-365.
2. Barykina NV, Subach OM, Doronin DA, Sotskov VP, Roshchina MA, Kunitsyna TA, Malyshev AY, Smirnov IV, Azieva AM, Sokolov IS *et al*: **A new design for a green calcium indicator with a smaller size and a reduced number of calcium-binding sites**. *Sci Rep* 2016, **6**:34447.
3. Shaner NC, Lambert GG, Chammas A, Ni Y, Cranfill PJ, Baird MA, Sell BR, Allen JR, Day RN, Israelsson M *et al*: **A bright monomeric green fluorescent protein derived from Branchiostoma lanceolatum**. *Nature methods* 2013, **10**(5):407-409.
4. Thestrup T, Litzlbauer J, Bartholomaus I, Mues M, Russo L, Dana H, Kovalchuk Y, Liang Y, Kalamakis G, Laukat Y *et al*: **Optimized ratiometric calcium sensors for functional in vivo imaging of neurons and T lymphocytes**. *Nature methods* 2014, **11**(2):175-182.
