# Supplementary material for: Genetically encoded calcium indicator with NTnC-like design and enhanced fluorescence contrast and kinetics
Source: BMC Biotechnol. 2018 Feb 13;18:10. doi: 10.1186/s12896-018-0417-2 (PMC5812234; doi:10.1186/s12896-018-0417-2)
Supplement: Supplementary file 10 — Table S2. Characteristics of calcium ions responses to intracellular stimulation with 10 APs in neurons expressing iYTnC2 and GCaMP6s sensors in dissociated neuronal culture. (PDF 12 kb) [file 12896_2018_417_MOESM10_ESM.pdf]

	Rise time $t_{1/2}$, ms [a]	Decay time $t_{1/2}$, s [b]	$\Delta F/F_0$ [c]	SNR [d]
GCaMP6s (n=7)	304±11	9.69±1.19	0.74±0.08	310±50
iYTnC2 (n=9)	258±35	6.64±0.9	0.16±0.03	134±37

[a] rise half-time was measured as a time between the stimulus onset and half-peak of response.

[b] decay half-time was calculated as a time from the peak to half-peak on the back front of response.

[c] $\Delta F/F_0$ was calculated as $(F-F_0)/F_0$, where F_0 is the baseline fluorescence signal averaged over a 1-s period immediately after the start of imaging.

[d] Signal-to-noise ratio (SNR) was quantified as the peak $\Delta F/F_0$ response over the standard deviation of the signal during a one second period before stimulation.

Mean values ± standard error of mean are given. $\Delta F/F_0$ and SNR values were normalized using $\Delta F/F_0$ measured for R-GECO1 response to 10APs in each recorded cell.
